# Behavior of Drinking Water Distribution Pipes Made of HDPE, AC, OLT 37, and OL37.1 in Contact with Water

**DOI:** 10.3390/polym18182186

**Published:** 2026-09-08

**Authors:** Daniela Simina Stefan, Georgeta Teodorescu, Adrian Ionut Nicoara, Lucretia Ghenghea, Ana Iulia Stefan

**Affiliations:** 1 Department of Analytical Chemistry and Environmental Engineering, Faculty of Chemical Engineering and Biotechnologies, National University of Science and Technology Politehnica of Bucharest, 1-7 Gh. Polizu Street, 011061 Bucharest, Romania; georgeta.teodorescu@upb.ro; 2 Department of Science and Engineering of Oxide Materials and Nanomaterials, Faculty of Chemical Engineering and Biotechnologies, National University of Science and Technology Politehnica Bucharest, 1-7 Gh. Polizu Street, 011061 Bucharest, Romania; adrian.nicoara@upb.ro; 3 National Research Center for Micro and Nanomaterials, National University of Science and Technology Politehnica Bucharest, 313 Spl. Independenței, Street, 060042 Bucharest, Romania; 4 S.A. Ecoqua Calarasi, 6-8, Eroii Revoluţiei 22 Decembrie 1989 Street, 910001 Calarasi, Romania; lucretiaghenghea@yahoo.com; 5 Regina Maria-Dental Hospital, 71 Cozia Street, 300425 Timisoara, Romania

**Keywords:** drinking water, water distribution network, pipes composition, asbestos cement, OLT 37, OL 37.1, HDPE, HDPE oxidation, chlorine degradation, SEM, EDX, FTIR

## Abstract

Drinking water is essential for humans; its quality determines the health and proper functioning of the body. In order to obtain drinking water in accordance with the legislation in force, the performance of the technology applied for water treatment and the infrastructure for transport and distribution to the consumer are equally important. Particular importance to this last aspect was given by the introduction of European Directive 2020/2184. Infrastructure can significantly influence water quality due to the fact that there are periods when water stagnates in the pipe and electro-corrosion processes, and salt deposits or degradation/corrosion of the pipes can occur, or periods when water circulates under pressure, when deposits but also particles from the pipes are mechanically detached and transported to the consumer. In this article, we aim to present the behavior of asbestos-cement pipes, AC, special steel for pipes OLT 37, galvanized steel, OLT 37.1, and high-density polyethylene, HDPE, in contact with drinking water. The study was conducted in Calarasi city, Calarasi county, Romania, where some of the old pipes were replaced. A comparative study of new pipes of the same type with pipes in use for more than 20 years, up to 46 years, was conducted, and at the same time the changes that the pipes (which have not yet been replaced) have on the quality of drinking water for street consumers were also analyzed. Following the analyses performed, it can be stated that the water quality is within the maximum permissible limits, but the micropollutants that appear can accumulate in the body (such as AC microfibers, microplastics, metallic zinc, zinc ions, iron ions, manganese, aluminum) with effects that can be evident after a long period of consumption through bioaccumulation.

## 1. Introduction

Water is a very important resource for the health of the human body. The production of drinking water under the conditions imposed by legislation in force is a permanent concern. Ensuring water quality depends on the quality of the water source, the performance of the technology applied, and the quality of the transport and distribution infrastructure to domestic and industrial consumers [1,2]. Particular attention was paid to the technology applied to obtain drinking water, and less to the transport and distribution infrastructure. Directive (EU) 2020/2184 of the European Parliament and of the Council of 16 December 2020 on the quality of water intended for human consumption (recast) makes explicit reference to the quality of materials that come into contact with water and to the potential to release potentially toxic substances into drinking water. Thus, it was mentioned that norms must be introduced to characterize pipes so that they do not pose a danger to the health of drinking water consumers [3]. Considering this directive, it is important to know the behavior of pipes in contact with water. One of the criteria according to which the choice of materials for drinking water transmission and distribution pipes must consider the type of material they are made of and the resulting corrosion products [4]. There is a wide variety of pipe materials have been used for the transport of drinking water [5], such as: asbestos cement prohibited [6], ductile iron pipes with a service life of more than 50 years [7], special steel for pipes [8], polyvinyl chloride with good corrosion resistance [9] high density polyethylene with corrosion resistance [10,11], concrete pipes with long service life [12], galvanized steel pipe [13] and other.

**Figure 1:**
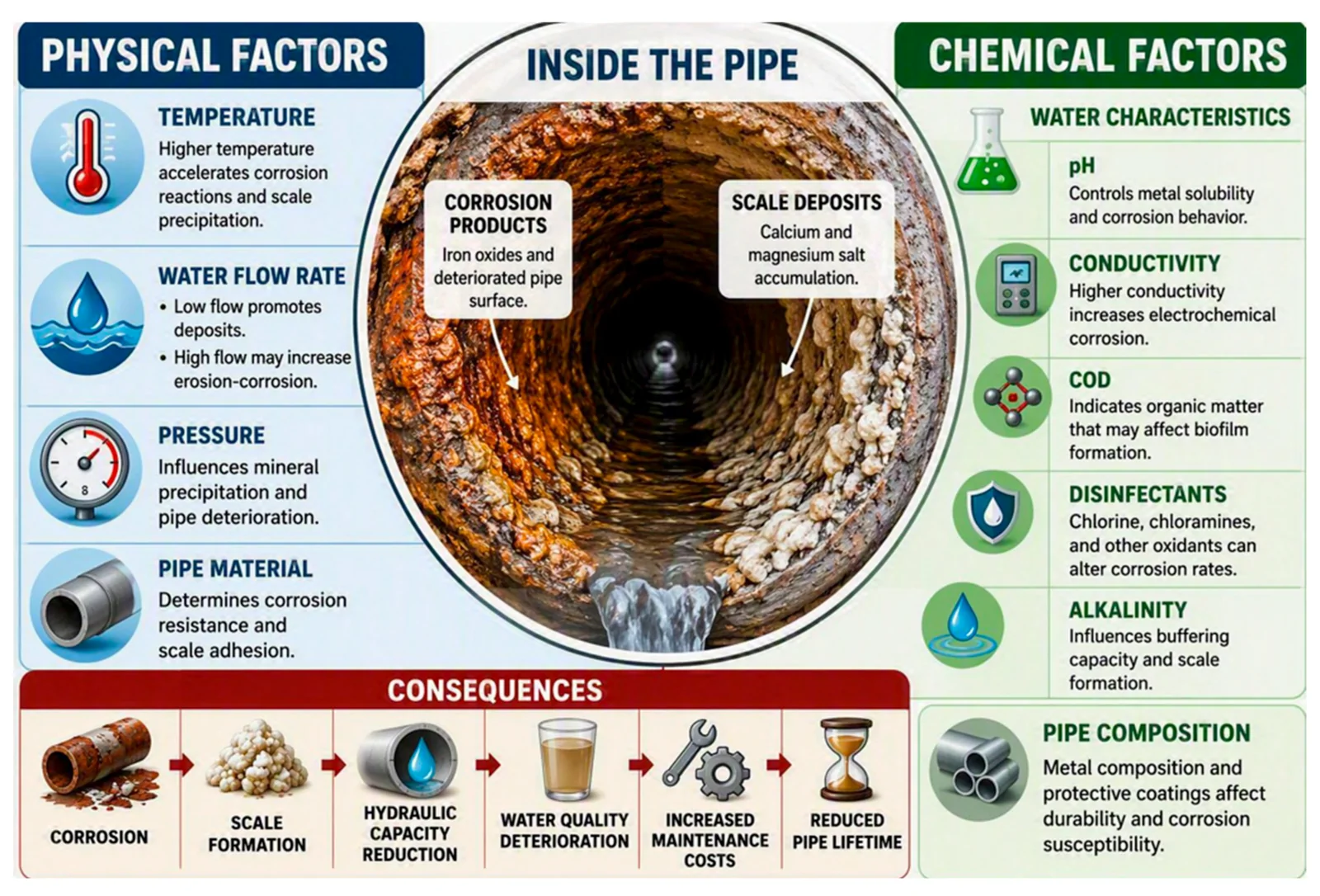
Factors influencing the corrosion process of water transport pipelines.

The processes corrosion or deposits, that take place in the pipe are influenced by the type of pipe material; water characteristics (pH, dissolved oxygen, hardness, oxidizability, conductivity, redox potential, organic compound content, disinfection substance, etc.); environmental conditions (day-night and winter-summer temperature variation, soil characteristics); operational factors (pressure, speed of water circulation in the pipes from periods of maximum consumption with maximum flow rate and minimum consumption with stagnation of water in the pipes) and others, see Figure 1 [14,15].

**Figure 2:**
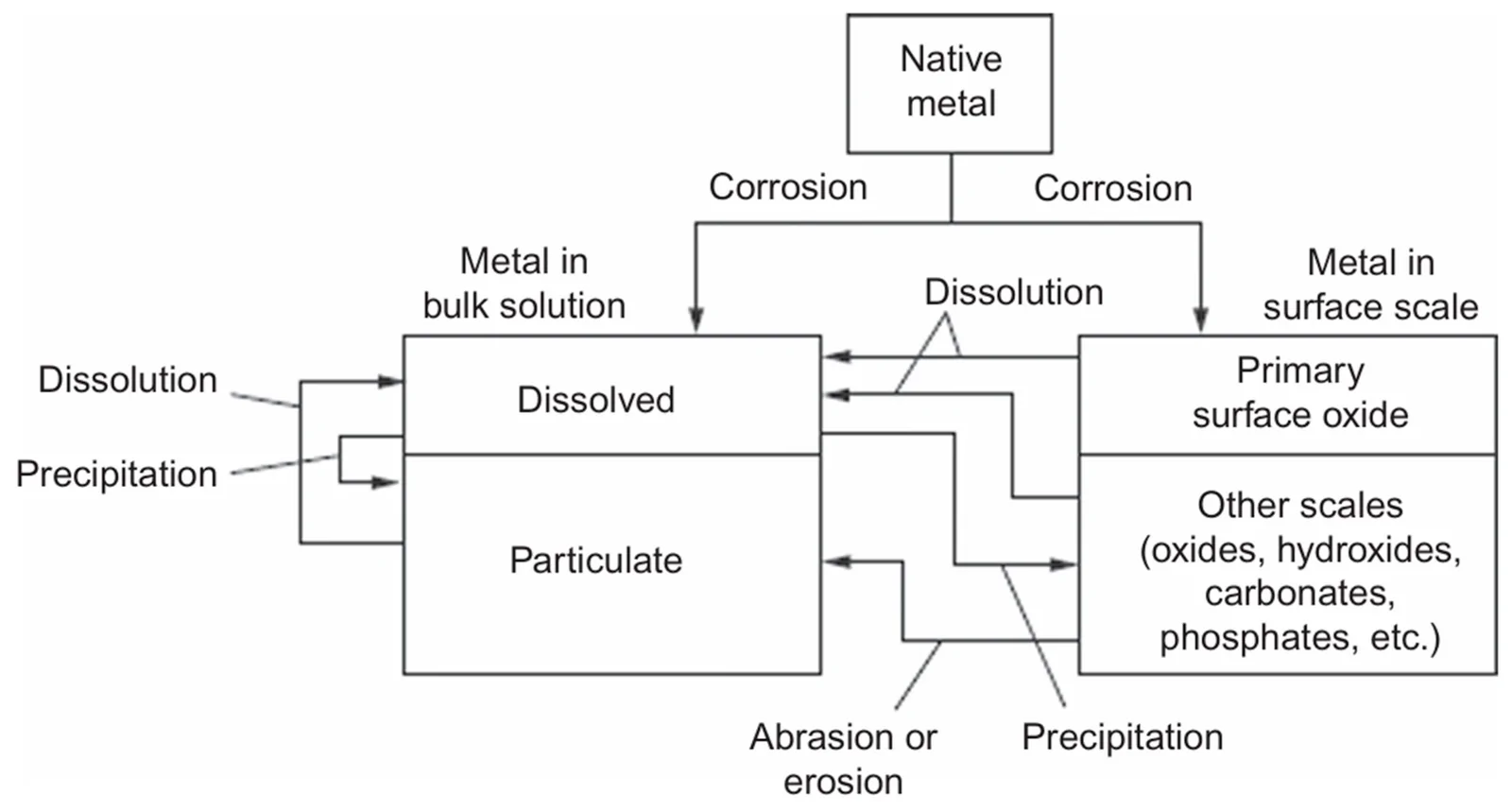
Schematic representation of the corrosion processes of conducting particle formation and salt deposition [16].

Figure 2 shows the main processes that can occur in a pipeline. Corrosion processes consist of the first stage of metal oxidation, its solubilization, and the formation of oxides and hydroxides that can pass into the water in the form of particles or soluble salts, causing an increase in turbidity or conductivity; in the second stage, the particles can settle on the surface of the pipe, forming crystals or amorphous substances. The processes are favored by the presence of oxygen in the water. During periods of stagnation of water on the bottom of the pipes, salts dissolved in water can concentrate. In contact with the pipe, they can adhere to the surface, interact with the pipe, or precipitate due to concentration, forming insoluble salts such as carbonates, sulfates, and hydroxides that can be deposited on the pipe or can be carried with the water current, also causing an increase in turbidity in the water.

Asbestos cement pipes, AC, can undergo processes of simple dissolution of cement products, deposition of carbonates, sulfates, hydroxides of calcium, magnesium, iron, or manganese, and aluminum silicates depending on the composition of the water, asbestos exposure, and release of asbestos fibers into the water [17].

In metallic pipes used for water transportation, corrosion processes occur due to dissolved gases such as oxygen, carbon dioxide, and hydrogen sulfide, resulting in oxides, sulfides, sulfates, and carbonates of bivalent and trivalent iron. Corrosion is stimulated by metallic impurities, bacterial microfilms, thermal variations, pressure variations, and water flow velocity [18].

Polymeric pipes of the HDPE, PVC, and LDPE type are vulnerable in the presence of chlorinating agents, especially chlorine oxide. Following the oxidation of methylene groups, carbonyl groups are formed, which leads to an increase in the carbonyl index. Following the attack, aldehydes, ketones, and acids are formed, which cause fragmentation of the polymer chains. Oxides, carbonates, sulfates, and microfilms can also be deposited on the surface of the pipe [19].

The research carried out aimed to identify structural modifications that occur in pipes used in water transport systems intended for human consumption and the effects that these pipes have on water quality. In this study, we studied how drinking water transport and distribution pipes made of asbestos cement, AC, OLT 37 steel, galvanized OLT 37 steel, and high-density polyethylene, HDPE, were corroded after a long period of use. In this regard, used and new pipes were comparatively analyzed, and we also studied how the pipe changed the water quality from the exit of the station to the street consumer after traveling through a pipe of a certain type and a certain period of use.

## 2. Materials and Methods

### 2.1. Characterization of Pipes Before and After Use

This research studied how the composition and appearance of pipes used in the drinking water transport infrastructure in Calarasi city, Calarasi county, changed after a long period of use from 20 to 46 years. It also studied how the old drinking water transport infrastructure influences the quality of drinking water for street consumers in the public domain.

The first drinking water transport system was built in Calarasi city between 1978 and 1980 and has been continuously developed until the present, in several stages. Therefore, pipes made of different materials such as asbestos cement, AC, OLT 37 steel, galvanized OLT 37 steel, and high-density polyethylene (HDPE) were used for water distribution. After a period of use ranging from 20 to 46 years, the old network began to be gradually replaced, mainly with high-density polyethylene pipes.

**Figure 3:**
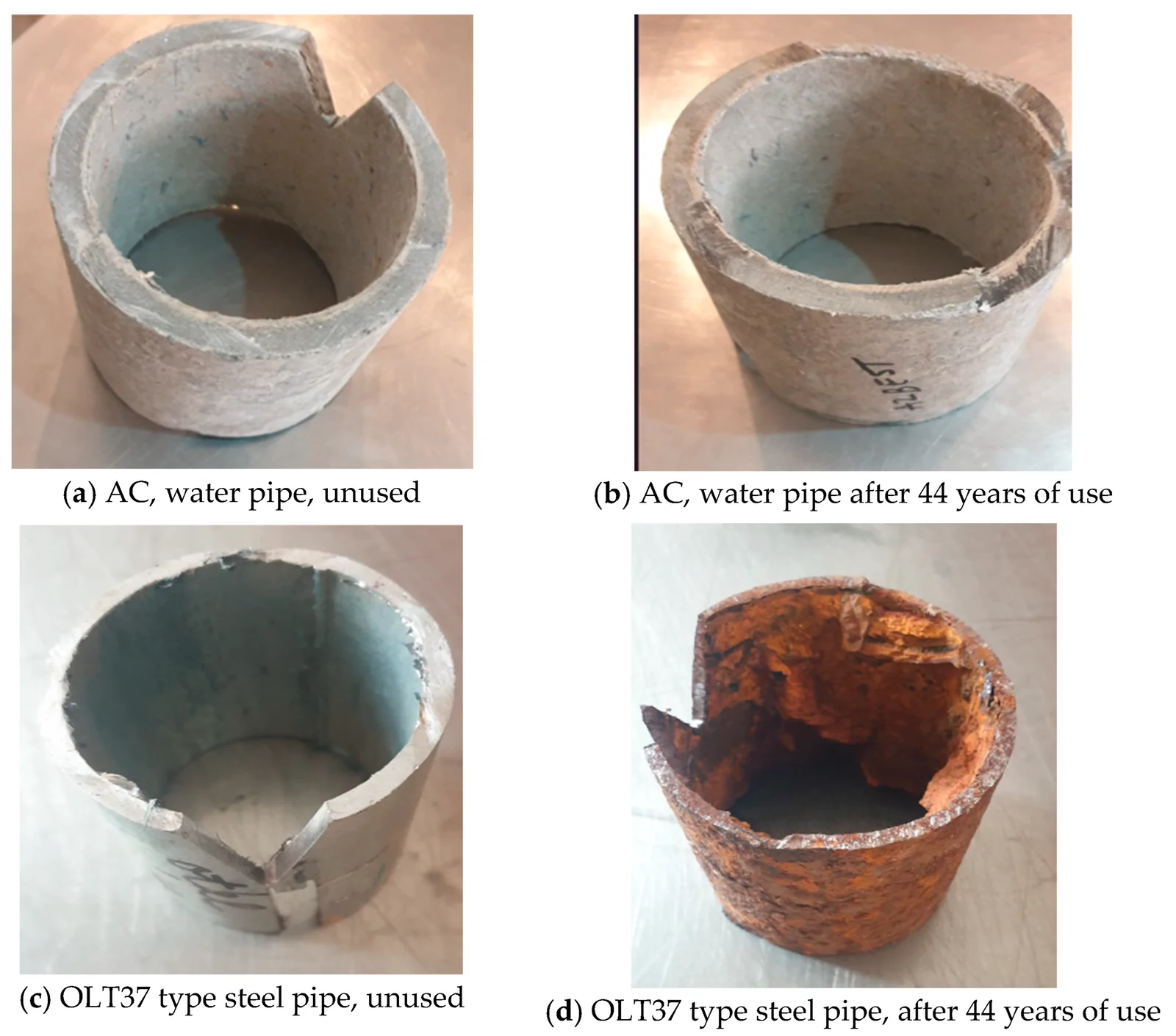

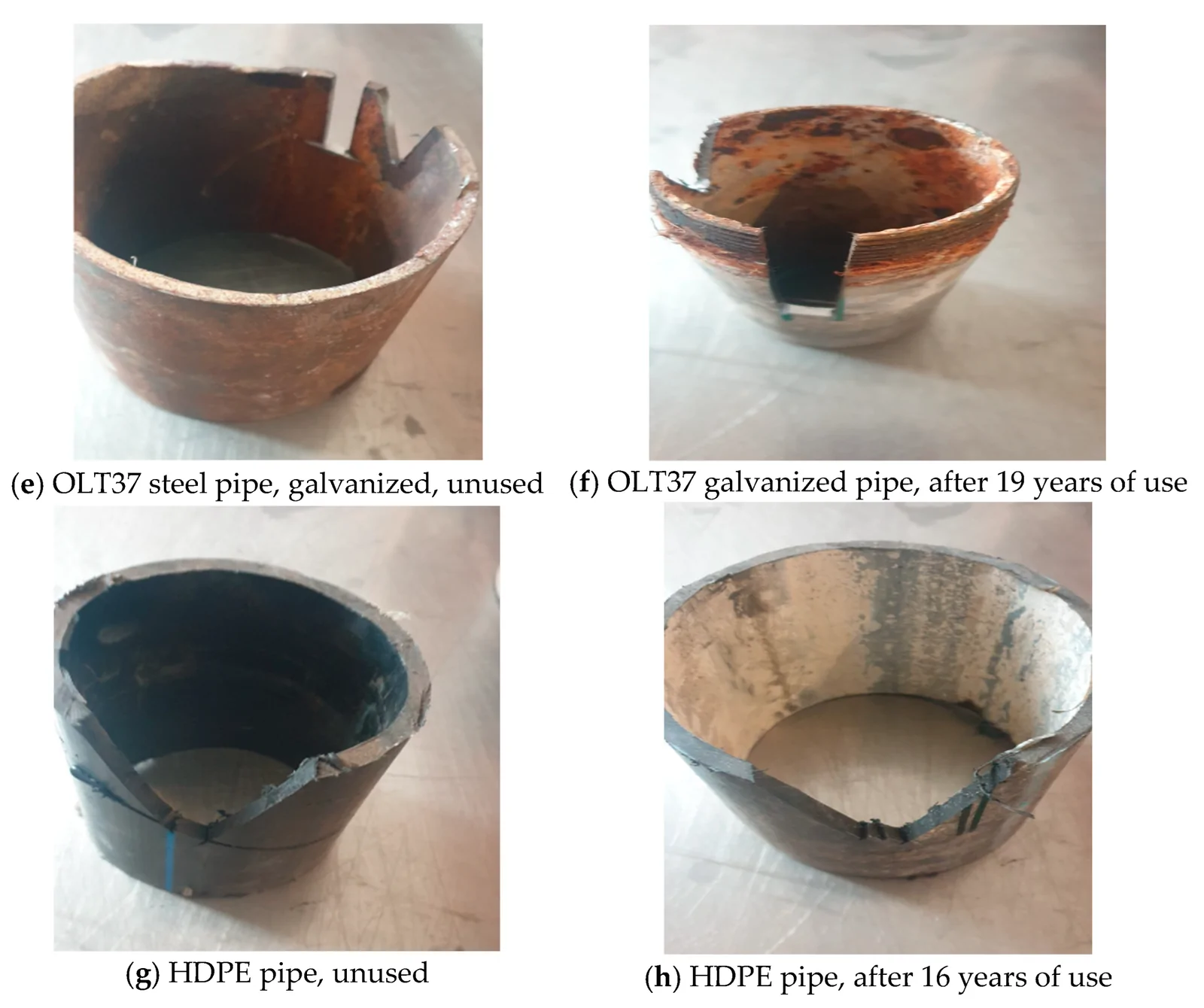
Images of new and used pipes used in the drinking water transportation infrastructure of Calarasi city.

The new and used pipes used in this study were provided by ECOAQUA S.A Călărași, Romania, the Regional Water and Sewerage Operator in Calarasi county. The pipes on which the study was carried out are presented in Figure 3.

Scanning electron microscopy (SEM) was used to examine the morphology and composition of the pipes before and after use. Energy-dispersive X-ray spectroscopy (EDX), combined with Scanning Electron Microscopy (SEM), was used to determine the elemental composition of the materials and deposition/corrosion products of the unused/used pipes. For this, a Quanta Inspect F50 (FEI Company, Eindhoven, The Netherlands), incorporated with an energy dispersive X-ray spectrometer, produced by EDAX (Mahwah, NJ, USA), consisting of Element EDS Analysis EDX GENESIS XM4 Software (Mahwah, NJ, USA).

The thickness of the new and used pipes in the minimum and maximum areas, with deposits, was measured using a caliper.

HDPE pipe material was subjected to FTIR analysis in the range of 4000 cm^−1^ and 100 cm^−1^, at a resolution of 4 cm^−1^. A Nicolet IS50FT-IR spectrometer equipped with a DTGS detector, produced by Nicolet, Wilmington, MA, USA, which provides information with high sensitivity, was used.

### 2.2. Analysis of Water Samples

In order to highlight the changes that occurred in the quality of water for the public consumer, water samples were taken from the Water Treatment Plant, WRP, which supplies the city of Calarasi, and from 4 points in the local distribution system, consumers located on the public domain, in order to avoid the influence of the pipes in the private supply system.

The sampling points were chosen so that the route along which the water moved contained asbestos-cement pipes, OLT 37 steel, Galvanized steel, OLT 37.1, and High-Density Polyethylene (HDPE). The samples were taken in the morning around 7 o’clock.

The parameter values were compared with the characteristics of the water at the outlet of the WTP-Calarasi Water Treatment Plant, Calarasi County. Table 1 presents the sampling points for drinking water samples. The experiments were carried out in three sampling campaigns in 2023 and 2025, namely: 21 September 2023, 22 August 2024, and 22 September 2025.

Sample collection, storage, and transport were carried out in accordance with the current standards: ISO 5667-5:2006; Water Quality—Sampling—Part, and ISO 5667-3:2024; Water Quality—Sampling—Part 3: Preservation and Handling of Water Samples. Samples were stored in glass containers.

**Figure 4:**
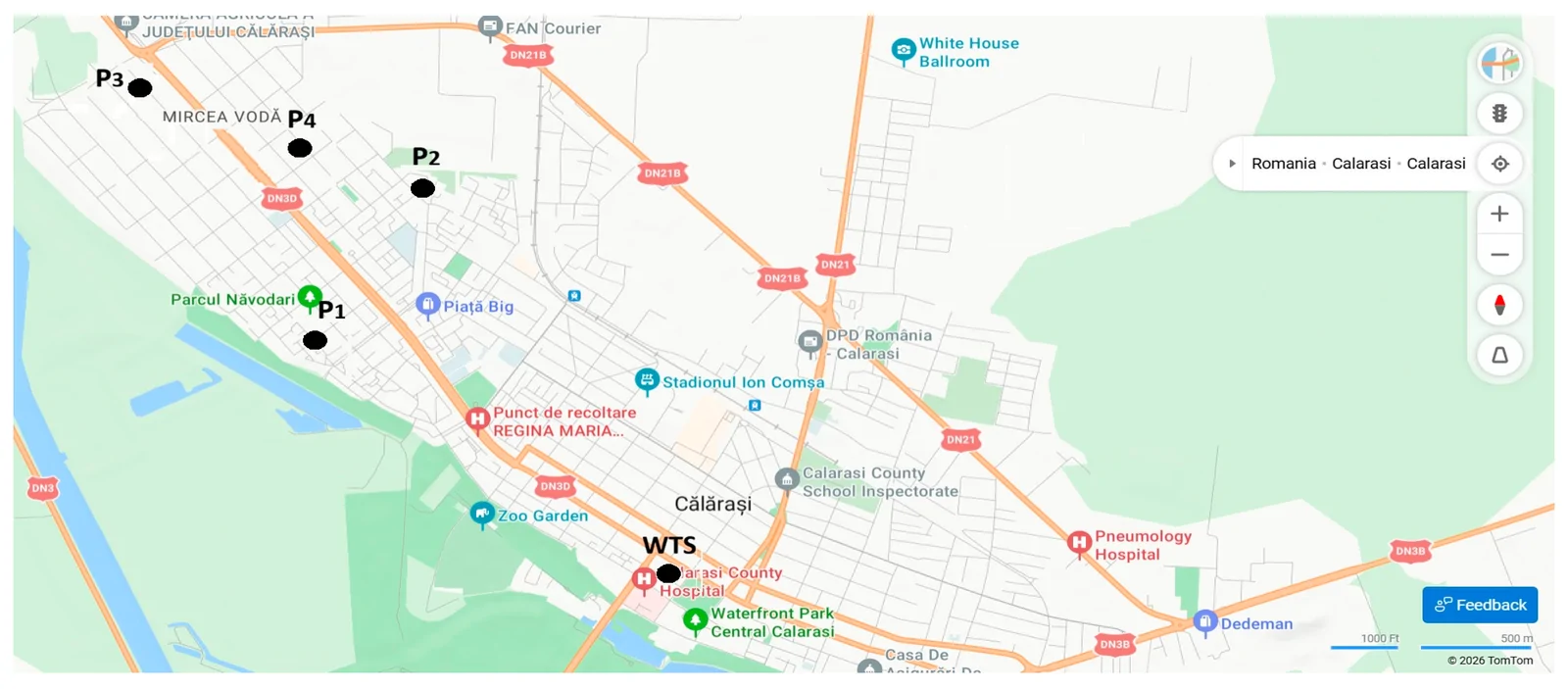
Position of the water sampling points on the Calarasi city Map, the points were added to the Calarasi Map taken from Map Romania, chart Bing Maps (Microsoft Corporation, Redmond, WA, USA) [20].

Figure 4 shows an image of the water sample sampling points on the Calarasi city map.

The parameters of interest that were monitored for the water at the outlet of the WTS were: pH, turbidity, conductivity, free residual chlorine, oxidizability (COD), ammonium, nitrites, nitrates, chlorides, sulfates, hardness, iron, manganese, aluminum, sodium, sulfates, sulfides, and for the water from the city network: pH, turbidity, conductivity, oxidizability—Chemical oxygen demand (COD), nitrates, iron, manganese, and aluminum.

The analyses were performed in accordance with Directive 2020/2184 on the quality of water intended for human consumption. The pH was determined using a pH meter, model 370 pH/mV-meter; conductivity was determined using a portable model 470 conductometer/TDS, 0–1999 mS range, manufactured by Jenway, Hong Kong, China.

Turbidity was determined using a HACH 2100P 150 turbidimeter, Hach, Loveland, CO, USA.

The concentrations of nitrates, nitrites, and sulfates were determined with a UV-VIS Spectrophotometer DR6000, Hach, Loveland, CO, USA. The solutions for the calibration curves for turbidity, nitrates, nitrites and sulfates were prepared from standard solutions as follows respectively: Stablcal Turbidity Standard Solution, 200 NTU, Nitrogen-Nitrate Standard Solution, 100 mg/L, as NO_3_^−^-N (NIST), Nitrogen-Nitrite Solution 100 mg/L, as NO_2_^−^-N(NIST), Sulfate Standard Solution, 1000 mg/L as SO_4_^2−^ (NIST) produced by Hach, Loveland, CO, USA.

Al, Fe, Mn, and Zn parameters were determined by the method of atomic emission spectroscopy with inductively coupled plasma, ICP-EOS, using an OPTIMA 5300 DV Perkin Elmer apparatus with a hydride generation system by flow injection FIAS 400, automatic sampler S10, produced by Agilent, Santa Clara, CA USA.

The water samples were digested with nitric acid, 70%, the water samples were concentrated 10 times by boiling. Calibration solutions were prepared by dilution using Multi-element Standard Reference Material, type Quality Control Standard 21, Perkin Elmer, for Fe, Mn, Zn, and Quality Control Standard 7A, Perkin Elmer, 100 μg/mL for Al.

The rest of the reagents used in the analysis were of quality for the analysis, provided by Sigma-Aldrich. Merck KGaA, Darmstadt, Germany.

In Table 2, the analyzed parameters, measurement uncertainties, and analysis methods are presented.

## 3. Results and Discussion

A comparative study of the morphology and surface composition of the internal surfaces of new and old pipes, after long-term use for the transport of drinking water, was carried out. SEM characterizations were performed at three magnifications: 200, 2000, and 10,000 times. EDX spectra show the main elements present on the surface and their share in the total elements.

### 3.1. Asbestos-Cement Pipes

Asbestos-cement pipes were introduced into the drinking water supply system of Calarasi city in 1978. After more than 46 years of use, most of them have been replaced. Now, there are only two pipes, each a few tens of meters long, that are to be replaced shortly. Their replacement was determined by the legislation in force. Asbestos-cement is a material that contains asbestos fibers, a fibrous silicate mineral, formed by thin and durable fibers, which are embedded in a mass of cement.

In the composition of asbestos-cement, there are oxides of silicon, aluminum, iron, calcium, magnesium, manganese, and others in very small, subunit proportions, such as sodium, potassium, titanium, and sulfur [21].

**Figure 5:**
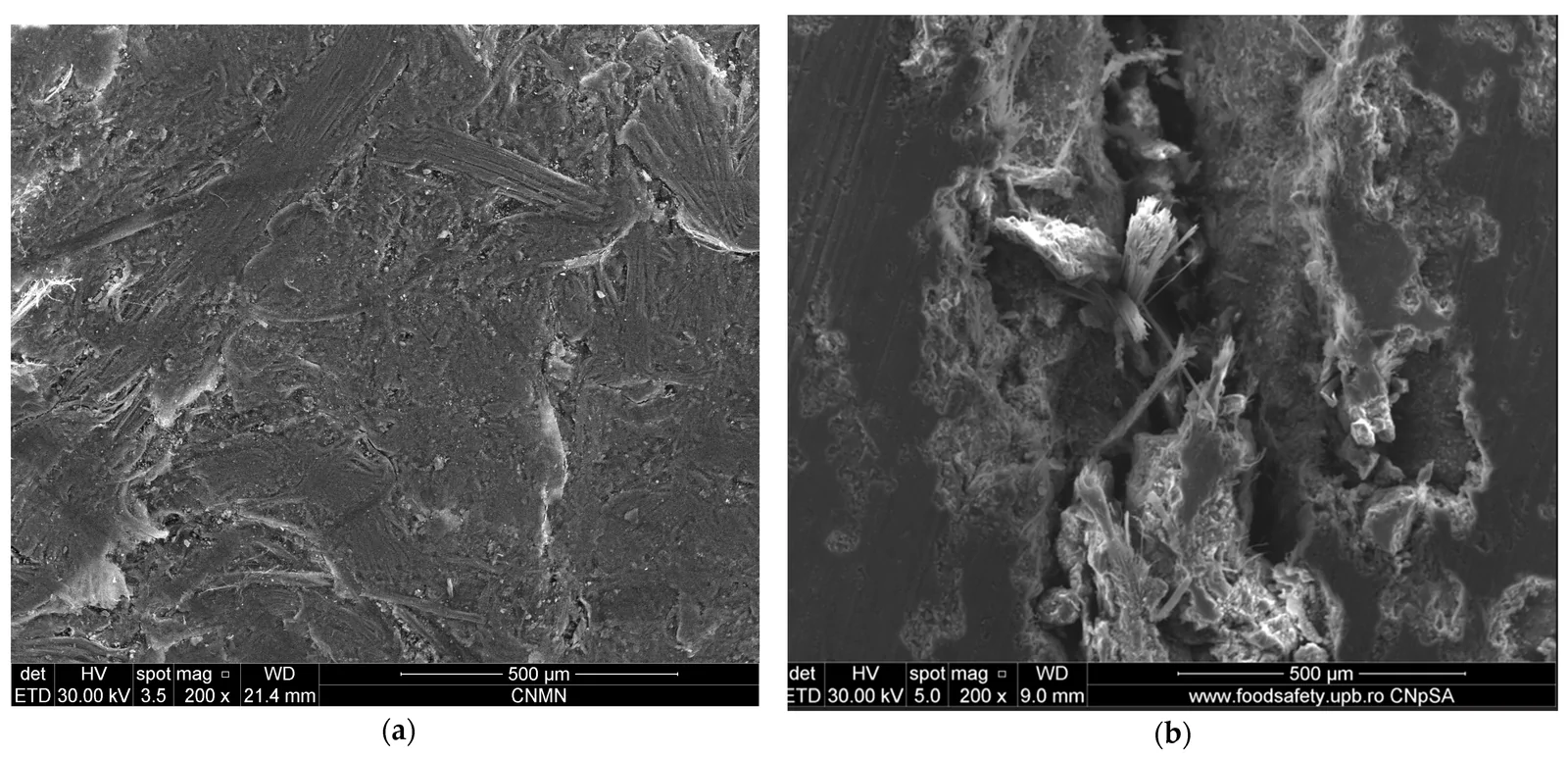

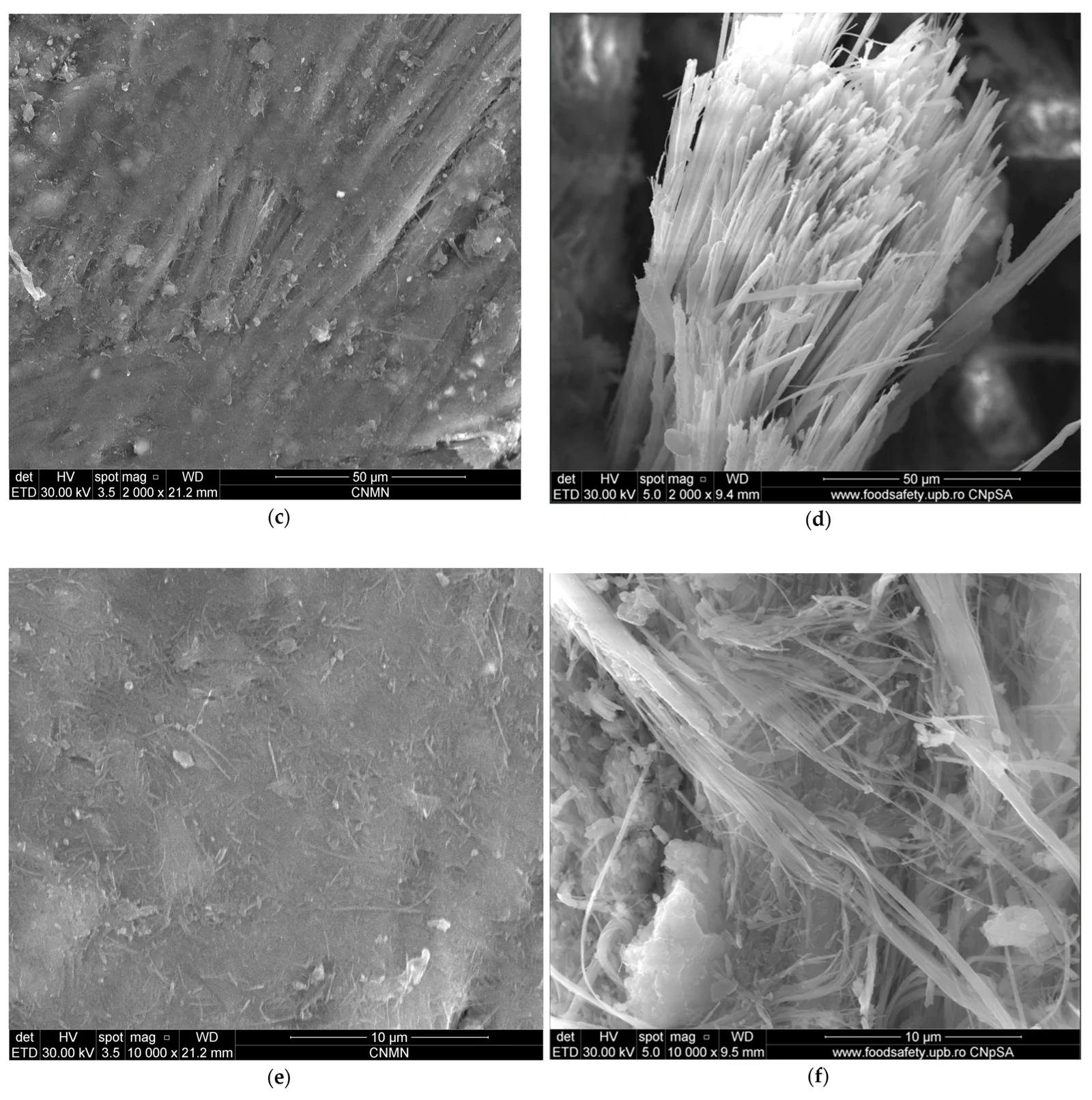
SEM analyses of the inner surface of asbestos-cement (AC) pipes, unused and used. (**a**) SEM of unused asbestos-cement pipe, magnification 200 times. (**b**) SEM of the used asbestos-cement pipe, magnification 200 times. (**c**) SEM of unused asbestos-cement pipe, magnification 2000 times. (**d**) SEM of the used asbestos-cement pipe, magnification 2000 times. (**e**) SEM of unused asbestos-cement pipe, magnification 10,000 times. (**f**) SEM of the used asbestos-cement pipe, magnification 10,000 times.

In order to highlight the changes suffered by the pipes, the wall thickness of the pipes was measured. The new and used pipes of the AC used in the evaluation were compared. The initial pipe thickness was 12 mm, and after 46 years of use, a thickness of 11.1 mm was identified. The changes that occurred were highlighted by SEM and EDX spectra (see Figure 5).

From the SEM Spectra analysis of unused asbestos-cement pipes, it can be observed that the inner surface has a uniform, smooth structure without lesions (Figure 5a,c,e). The asbestos mineral fibers are well incorporated into the cement. After 46 years of use, the inner surface of the pipe is corroded and uneven; the cement that embedded the asbestos has degraded and ground; lesions have appeared, and asbestos fibers of different sizes are visible (see Figure 5b,d,f). The danger represented by these pipes consists of particles of various oxides that can dissolve in water but especially can be found in the solid state as particles, causing an increase in turbidity and the content of dissolved salts. Numerous studies have been mentioned in the literature suggesting that these fibers have a special toxicity, causing cancer in the respiratory and digestive systems, and other cardiovascular diseases [22].

**Figure 6:**
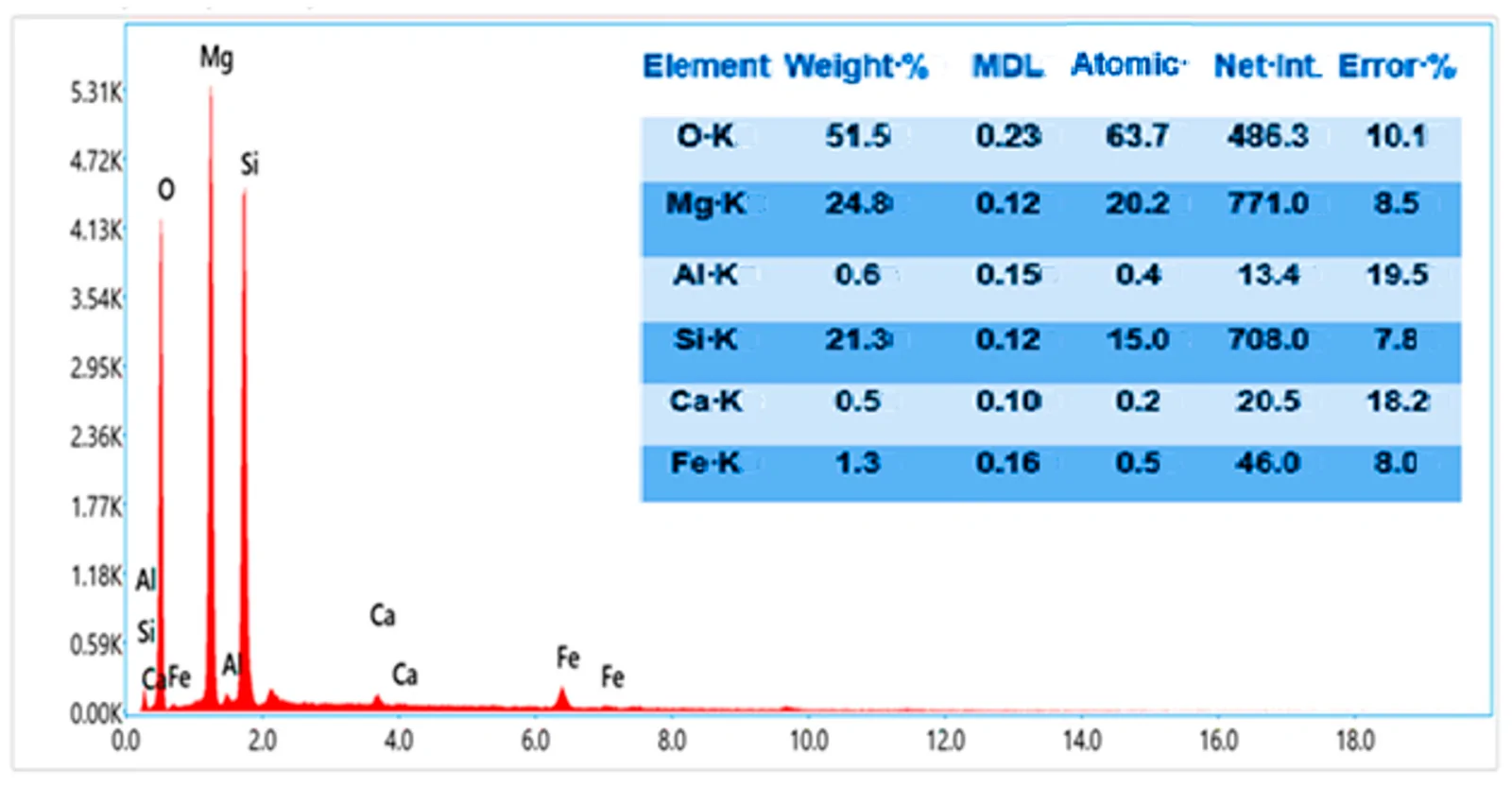
EDX spectrum of the inner surface of the unused asbestos-cement pipe and the mass percentage of the main elements identified.

The EDX spectrum of the unused AC pipe is shown in Figure 6. It shows the characteristic peaks of the elements found on the inner surface of the pipe. The mass and atomic percentages of the elements are presented in the attached table. Oxygen is found in the highest proportion, over 51% by mass. This suggests that the main constituents of the pipe are oxides, especially magnesium and silicon oxides, which are present in mass concentrations of over 20%. Small concentrations of aluminum, calcium, and iron oxides also appear. Similar results are also mentioned in the literature reference [21].

**Figure 7:**
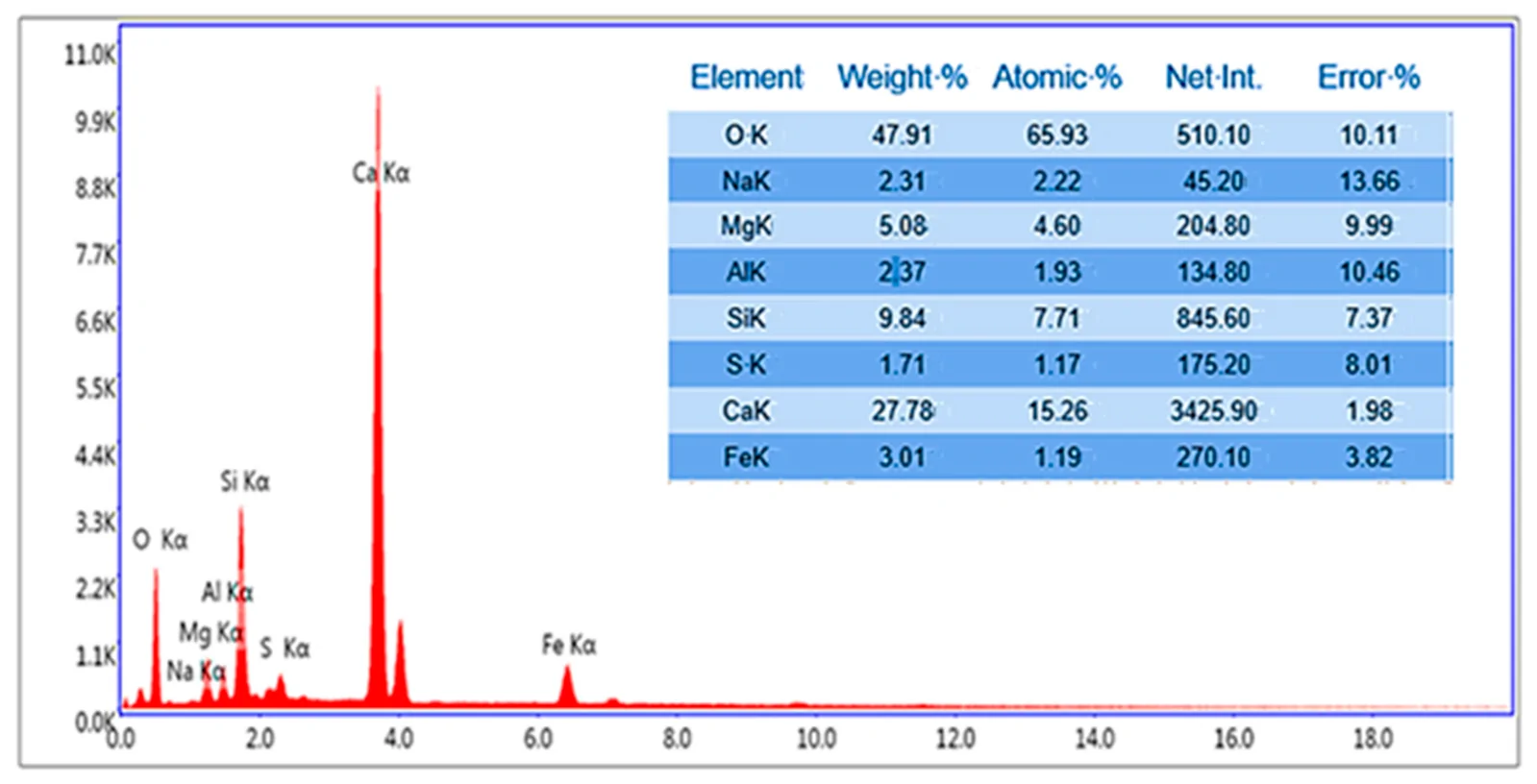
EDX spectrum of the outer surface of the asbestos-cement pipe used and the percentage of the main elements identified.

In the EDX spectrum of the used pipe, as shown in Figure 7, the ratios between the elements have changed significantly: the oxygen content has decreased to 48%, the magnesium content from 24.8% to 5.08%, and the silicon content from 21.3% to 9.84%. In return, the aluminum content has increased from 0.6% to 2.37%, the iron content from 1.3% to 3.01%, and the calcium content from 0.5% to 27.78%. New elements have also appeared, such as sodium in a proportion of 2.31% and sulfur in a proportion of 1.71%.

This composition suggests that calcium compounds, such as calcium carbonate; aluminum compounds, such as sulfate; aluminum oxides; and iron compounds, such as oxides or carbonates, have been deposited on the pipes.

Correlating the data obtained with the characteristics of the water at the exit of the station, but also at the sampling point P1, the street consumer on Nufarului Street (see Table 3), we observe that it has a hardness of around 9 °G (German hardness degree—10 mg CaO/L). So, the water has a high calcium and magnesium content.

The pH of the water varies in the weakly basic range, reaching in some periods around 7.5 at the entrance to the station and almost 8.00 at the sampling point on 22.09.2025. This pH value indicates a high alkalinity of the water, which can be mainly given by bicarbonates, carbonates, and hydroxides of sodium, potassium, calcium, and magnesium [23]. Therefore, it is possible that during periods of stagnation of the water, calcium is deposited in the form of calcium carbonate. Calcium oxides present in cement, when they come into contact with water, can form calcium hydroxide, which will cause a local increase in the pH on the pipe surface to values close to the calcium carbonate precipitation value of 8.3–8.4. Thus, it accumulates on the surface. In Figure 7, an unidentified peak appears to the left of the oxygen peak in the range of 0.25–0.35 Kev that can be attributed to carbon. Therefore, this scenario is possible.

In the analyzed water samples, we observe that the turbidity tends to increase from the station outlet by more than 0.150 NTU at the sampling point, P1. This parameter shows that particles found in the consumer’s water are constantly suspended in the pipe. These can be components of cement, calcium oxides, silicon, magnesium, or asbestos fibers, as Figure 5b,d,f show SEM images of the pipe used. These compounds, although not found in very high concentrations, can still create health problems for the consumer by accumulating over time. The literature reports that in the UK, more than 1,000,000 chrysotile asbestos fibers per liter of drinking water have been identified, with more of these in the size range of 1.4 and 0.04 µm [24].

We also observe that there is an increase of over 200 µS in conductivity, which proves that ions are released from the pipe. These ions can be iron ions (bivalent or trivalent), magnesium, silicon in the form of silicic acid, etc.

Another very important parameter whose variation is significant is oxidizability. This parameter indicates the amount of organic matter and reduced compounds present in the water. We observe that this parameter increases in the water at the consumer compared to the exit from the station, with values around 0.4 mg O_2_/L. This means that either organic matter accumulates in the water or the concentration of reduced inorganic species such as bivalent iron, sulfides, etc., increases. Residual aluminum has a decreasing concentration because it precipitates in the form of aluminum hydroxide, which explains the decrease in its concentration in the water.

The processes that occur at the water level and at the contact surface with the pipe are complex and antagonistic processes. During periods of water stagnation, partial dissolution of the pipe occurs, especially calcium oxide (quicklime), which causes a local increase in pH. This generates deposits of salts such as calcium carbonate (pH 8.3–8.4); aluminum hydroxides (pH 6–8.5); and iron hydroxide (pH 8–9) or iron sulfides under anoxic conditions in the possible presence of sulfur-reducing bacteria, organic compounds, and a pH between 6 and 8 [25]. When water circulates according to the consumption flow rate and pH, many of the salts deposited during the night are detached from the pipes and entrained as such, forming fine particles in the water. Another part of them, and part of the pipe, dissolves as iron hydroxide, silicon oxide, and some of the particles, and we find them in the form of conductivity in the water.

In conclusion, the pipe influences the quality of water for the street consumer. The possible danger is probably determined by the presence of asbestos particles, aluminum hydroxide, and other dissolved ions in the pipe and their concentration during the night in the pipe and their entrainment in higher concentrations during the day, in the first hour, immediately after opening the tap, but also over time throughout the day. The pipe grinds and dissolves, generating additional turbidity and dissolved salts, some of which are toxic.

### 3.2. Steel Pipes OLT 37

OLT 37 is rolled steel, special alloy, equivalent to OL 37 steel (STAS 500-89/1989), European steel S235 (EN 10025 2), and the international standard ASTM A36-A 36 M. It is commonly used for metal construction, fluid transport pipelines, and other things. Normally contains iron, Fe, with a concentration between 98.5 and 99.2%; carbon, C, in a concentration between 0.12 and 0.20%; silicon, Si, between 0.17 and 0.37; and other elements with low content such as Mn, P, S, Cr, Ni, Cu, etc. [26,27].

Following the measurement of the thickness of the OLT37 pipes, a variation in the diameter was found, from 4 mm initially to values of 3 mm in areas without deposits, with corrosion, and up to 12 mm in areas with deposits, after 42 years of use. The changes that occurred were highlighted by SEM and EDX spectra.

**Figure 8:**
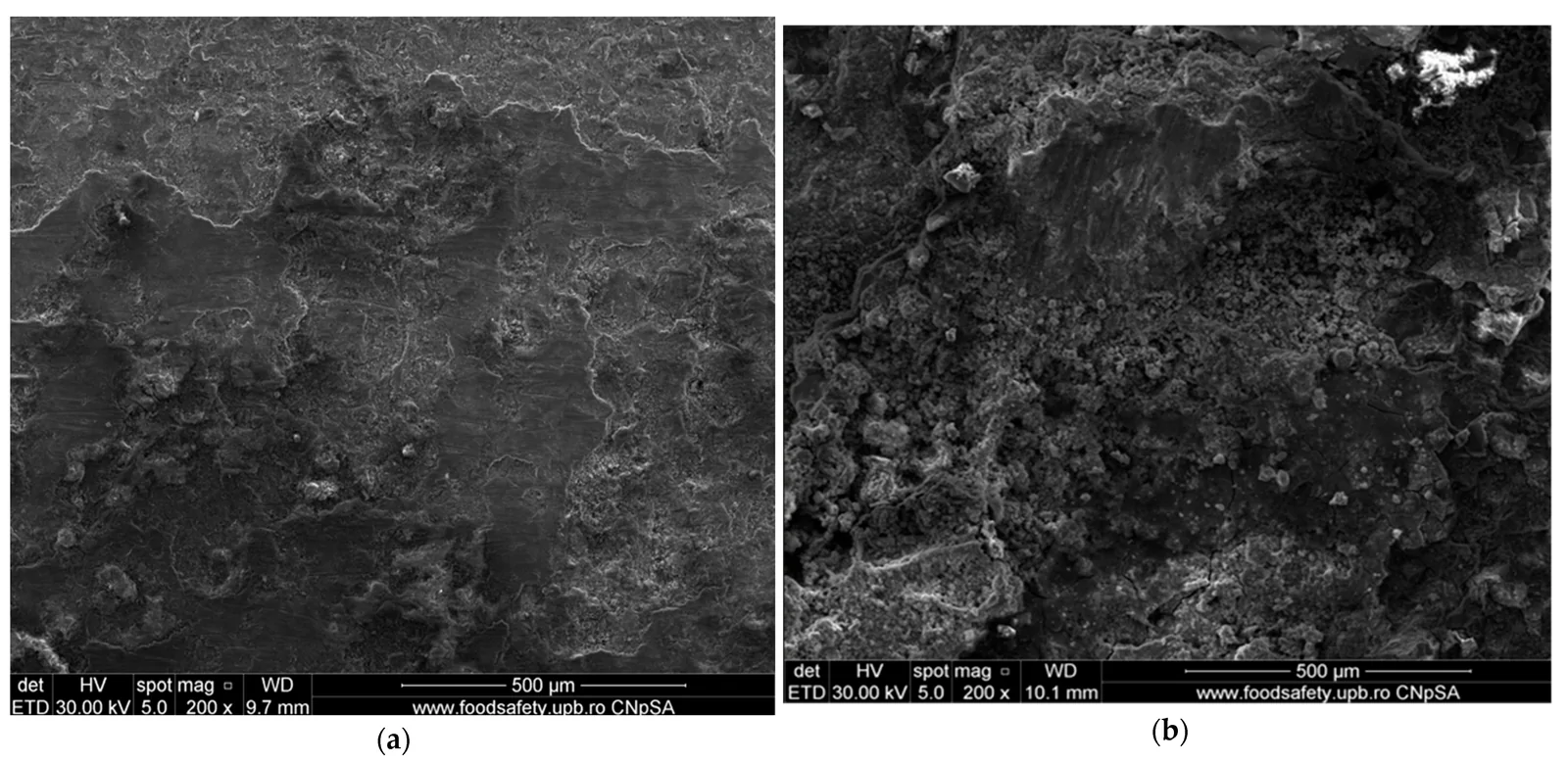

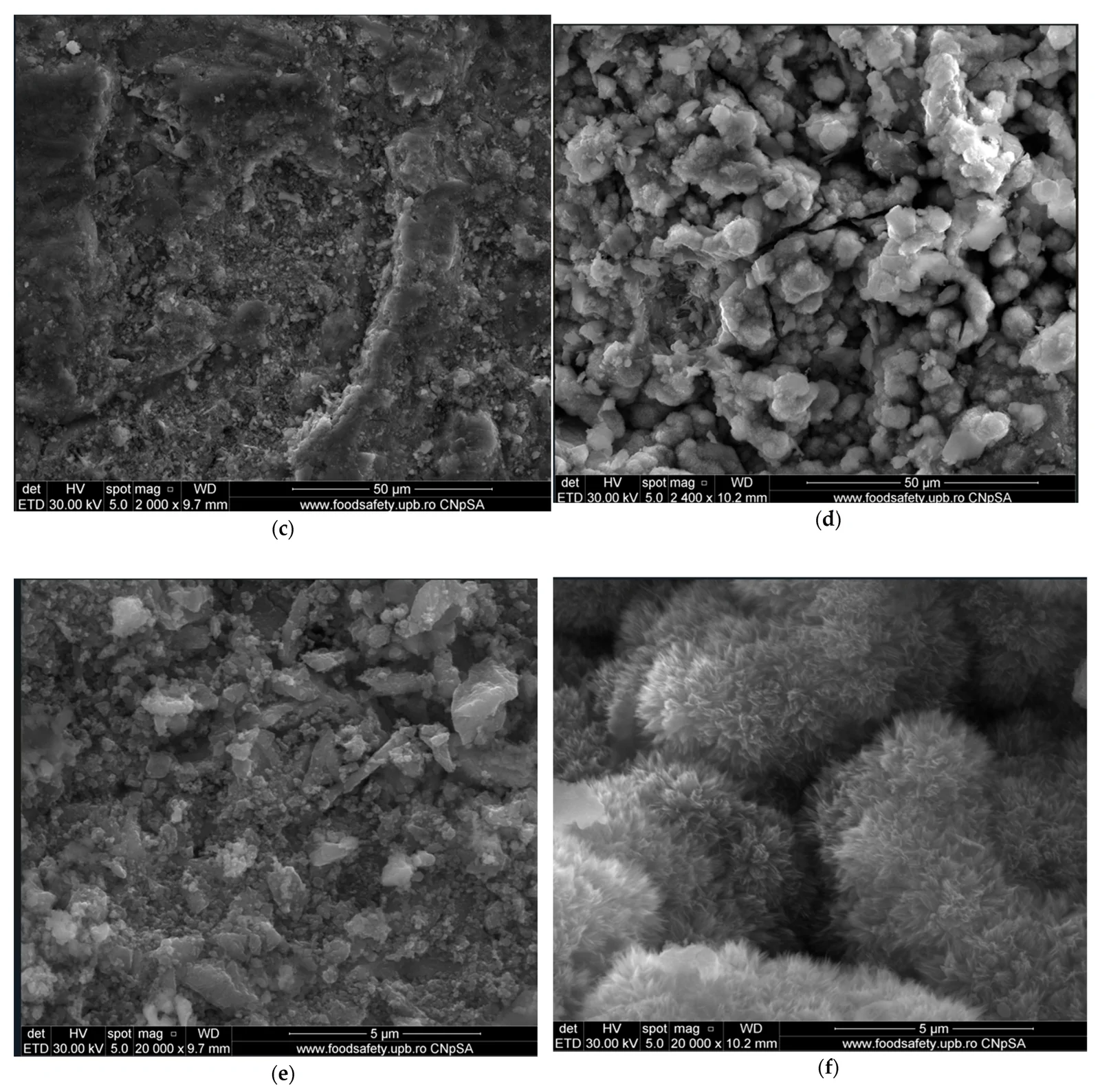
SEM analysis of the inner surface of OL37 rolled steel pipes, unused and used. (**a**) SEM of the OLT37 pipe, unused, magnification 200 times. (**b**) SEM of the OLT37 pipe, used, magnification 200 times. (**c**) SEM of the OLT37 pipe, unused, magnification 2000 times. (**d**) SEM of the OLT37 pipe used, magnification 2000 times. (**e**) SEM of the OLT37 pipe, unused, magnification 20,000 times. (**f**) SEM of the OLT37 pipe used, magnification 20,000 times.

**Figure 9:**
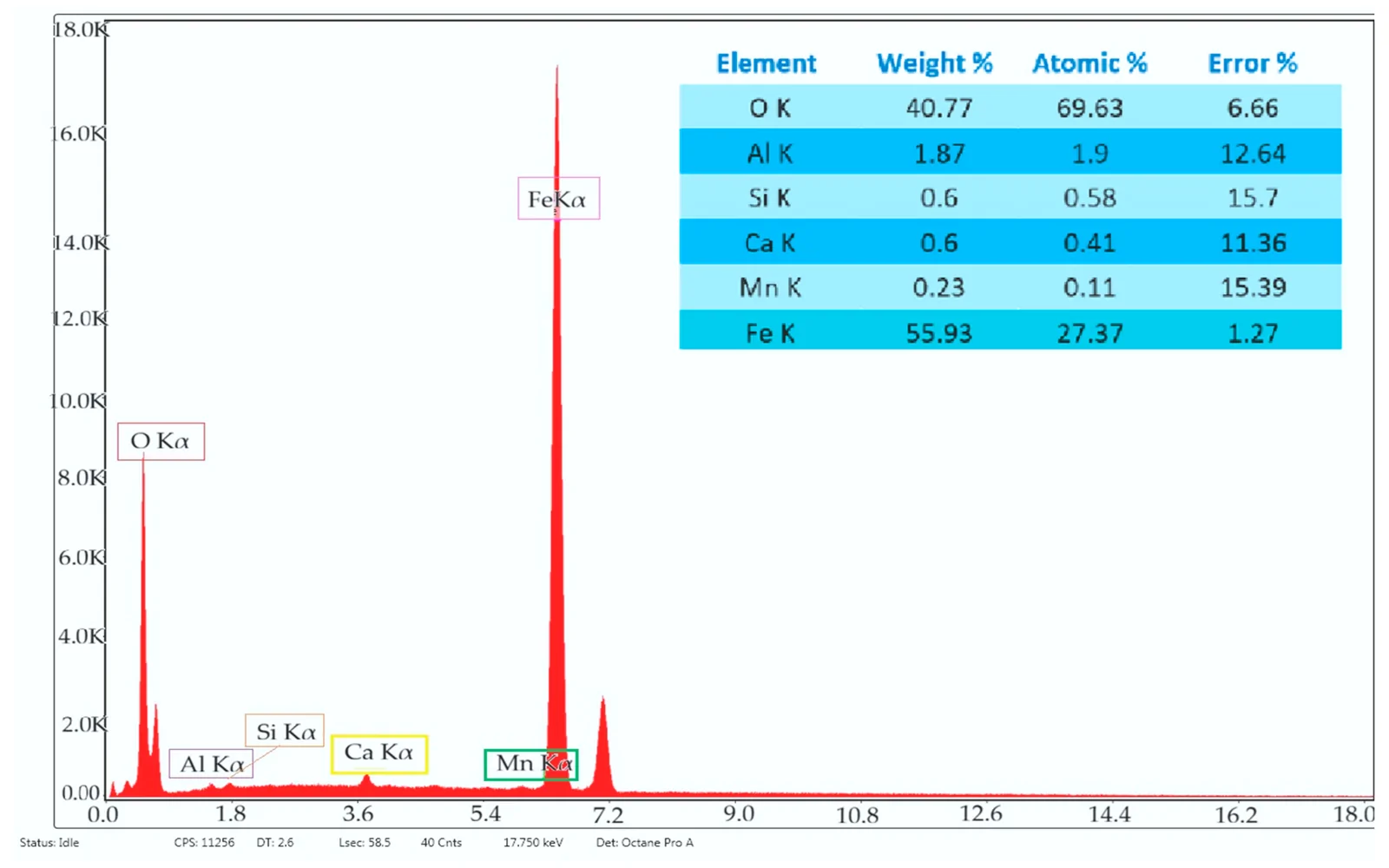
EDX spectrum of the inner surface of the unused OLT 37 pipe and the percentage of the main elements identified.

From the SEM images of the unused OLT 37 pipe at the three magnifications 200, 2000, and 10,000 (Figure 8a, Figure 8c and Figure 8d, respectively), it can be seen that the surface is relatively uniform and smooth, with some corrosion, without significant deposits. The chemical composition in the corroded region shows corrosion caused by water and atmospheric oxygen. From the EDX analysis and the surface composition, aluminum, iron, and silicon oxides appeared on the inner surface of the pipe (see Figure 9). Iron decreased from high concentrations of 98–99% to concentrations of approximately 56%, and oxygen appeared at concentrations of over 40%. Carbon is present in very low concentrations; the characteristic peak is to the left of the oxygen peak, which was not highlighted due to the very low content on the oxidized surface. Aluminum, silicon, calcium, and manganese ions were also identified with concentrations between 0.23 and 1.87%. This behavior of the initial pipe proves that these pipes are vulnerable to water and oxygen from the air but also from water.

**Figure 10:**
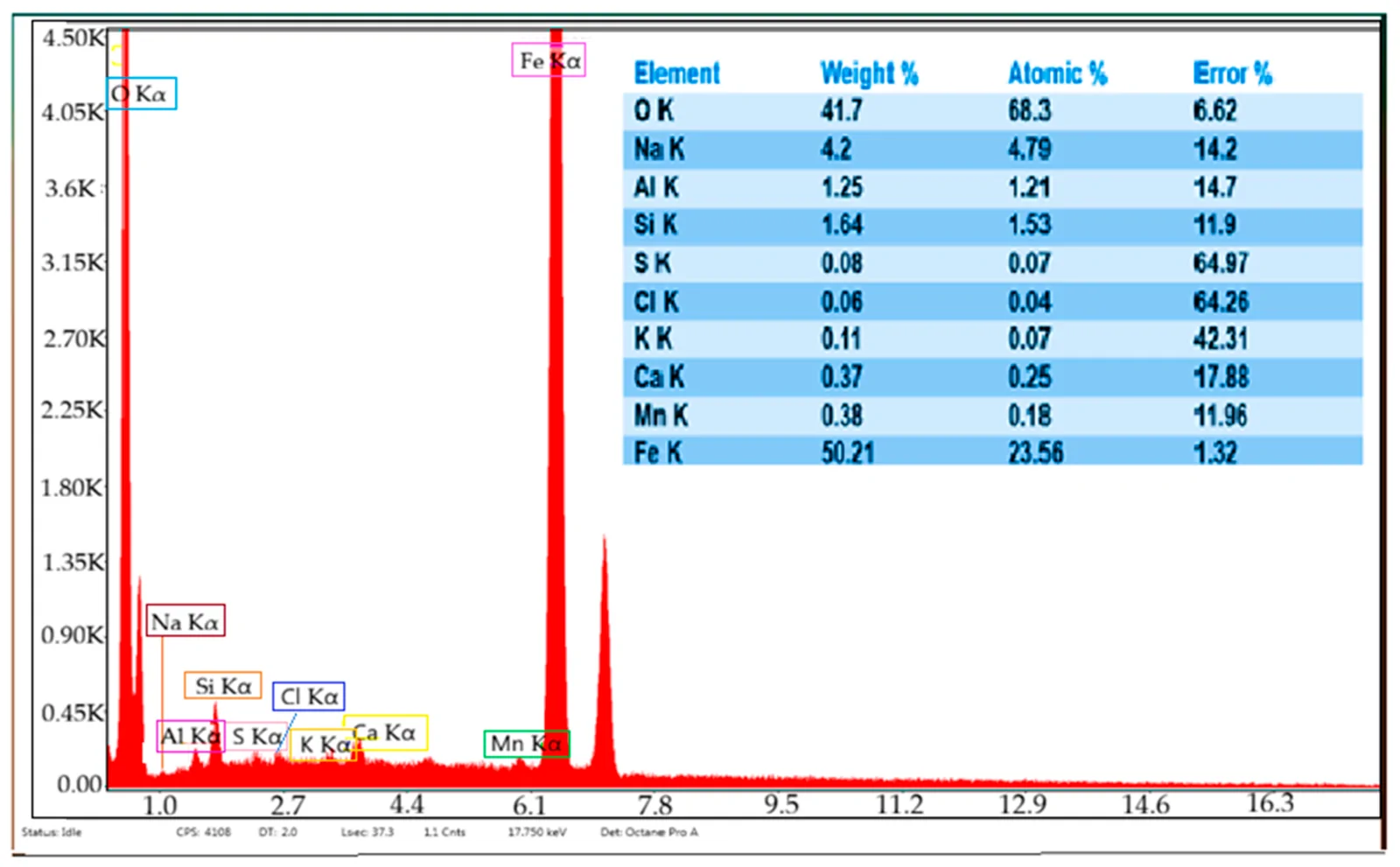
EDX spectrum of the inner surface of the used OLT 37 pipe for 42 years and the percentage of the main elements identified.

The inner part of the used pipe has a totally modified structure compared to the control pipe (see Figure 8b,d,f). Deep corrosion processes occur; the surface shows significant unevenness. The main elements present on the pipe surface are Iron (over 50%), oxygen (over 41%), and the remaining elements at low concentrations: Na, Al, Si, K, S, Ca, and M, according to Figure 10. Uniform crystal deposits appear in the cavities, such as aluminum hydroxide Al(OH)_3_, which can form at pH values between 6 and 9 [25]; Fe(OH)_2_, which can form at pH values higher than 8 [25]; and Mn(OH)_2_ at pH values higher than 8.5 and 9 [28]. Aluminum oxide is stable at pH values between 4 and 9; manganese oxide MnO_2_ at pH between 7 and 8; mixed oxides FeOOH and MnO_2_ [28]. Calcium carbonate, CaCO_3_, is formed at pH values > 8.3; manganese carbonate at pH values greater than 8.5 [24]; iron sulfides at pH values between 4 and 12 [28]; manganese sulfides, MnS, at pH between 4 and 12 [29]; calcium sulfate can also be formed [30,31]. These deposits were formed especially during the period of water stagnation following electrochemical processes, with the iron ion playing an essential role. In the first stage, the oxidation of metallic iron to trivalent or bivalent iron was achieved after the reaction:

In aerobic conditions

Anode (pipe)

Fe(s) → Fe^2+^ + 2 e^−^ (1)

Cathode (water):

O_2_ +2H_2_O+4e^−^ → 4OH^−^ (2)

In aerobic conditions, metallic iron from the pipe is oxidized; the electrons that are formed migrate to the cathodic areas, where they reduce oxygen, causing the formation of precipitates of iron hydroxide ions. The hydroxyl formed in the presence of bicarbonate ions can also react with other metal ions present in the water, such as manganese and calcium, forming deposits of CaCO_3_ and Mg(OH)_2_, etc. Oxygen can react directly with iron ions and other ions on the pipe surface, forming solid deposition oxides like Fe(OH)_3_, FeOOH, Fe_2_O_3_ [32].

In anaerobic conditions:

Anode (pipe)

Fe(s) → Fe^2+^ + 2e^−^

Cathode (water):

2H_2_O+2e^−^ → H_2_+2OH^−^ (3)

As a result of this reaction, deposits of Fe(OH)_2,_ Fe_3_O_4_, FeS, CaCO_3_, and Mg(OH)_2_ may appear on pipes [33].

These deposits protect the pipe from corrosion but also reduce the useful diameter of the pipe.

The characteristics of the water are particularly important in contact with the steel pipe; see Table 4. The pH is a decisive factor in the formation of various forms of precipitates. The pH of the water at the outlet of the station is between 7 and 7.5, and at the street consumer Strada Belsugului, point P2, it increases only very slightly, remaining in the same range of 7–7.5. It is observed that turbidity has a clear tendency to increase by 0.08 NTU, values that can be due to the precipitates mentioned above, traces of rust, and other compounds. There is a possibility that the particles that come off the pipe, together with the aluminum ions, may form coarse particles that are deposited in the pipe in elbow areas or at pipe joints. The conductivity of the water increases from the control, the exit from the station, to the sampling point, which proves that part of the ions in the pipe dissolves, namely iron and manganese ions, and are found mainly in reduced form because the oxidizability increases, sometimes tripling, which suggests an intense degradation of the pipe. The analyses of iron and manganese ions show that the concentration of iron and manganese increases in the water at the street consumer in P2. The aluminum ion decreases in the system; there is the possibility of its removal by coagulation with rust particles and other precipitates. The hardness decreases slightly, but this suggests that part of the calcium disappears from the system by precipitation in the form of calcium carbonate, sulfate, or sulfide, depending on pH and redox potential.

The water at the street consumer at point P2 meets the drinking water requirements, but the problems are determined by turbidity and the concentration of iron, aluminum, and manganese ions. The increase in turbidity is determined by the various precipitates of iron, manganese, calcium, and aluminum ions. These can bring additional toxicity by accumulating these dissolved ions and particles.

### 3.3. Galvanized OLT 37 Pipes

Pipes made of OLT 37 Galvanized, symbolized by OLT 37.1, contain OLT 37 on which a thin layer of metallic zinc has been deposited on the internal surface. Zinc, in an oxidizing environment, is passivated by forming oxides, thus preventing corrosion from contact with water and increasing the service life of the pipes. These pipes contain iron (Fe) in a mass concentration of around 99%, carbon (C): 0.2%, manganese (Mn): 0.8%, phosphorus (P): max. 0.06%, sulfur (S): max. 0.06%, according to Steel Grades OL37.1 [34]. The zinc layer can have varying thicknesses ranging from 55 to 200 µm, according to the EN ISO 1461:2022 working standards, and can have the total mass of zinc applied on both sides of the material varying depending on the manufacturer: Z200 (200 g/m^2^), Z275 (275g/m^2^), or Z450 (450 g/m^2^). In our case, the unused pipe falls into the Z275 category.

**Figure 11:**
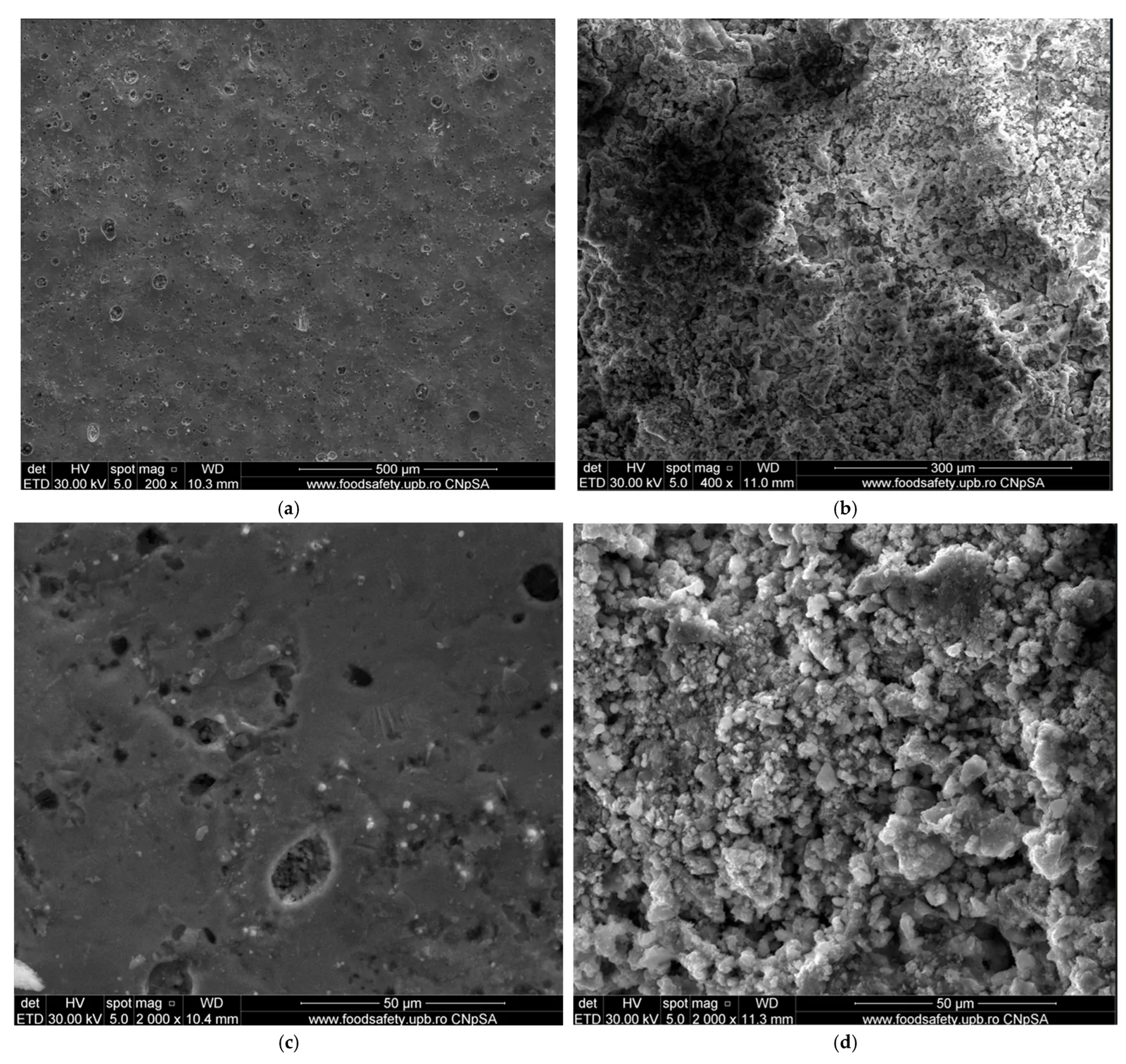

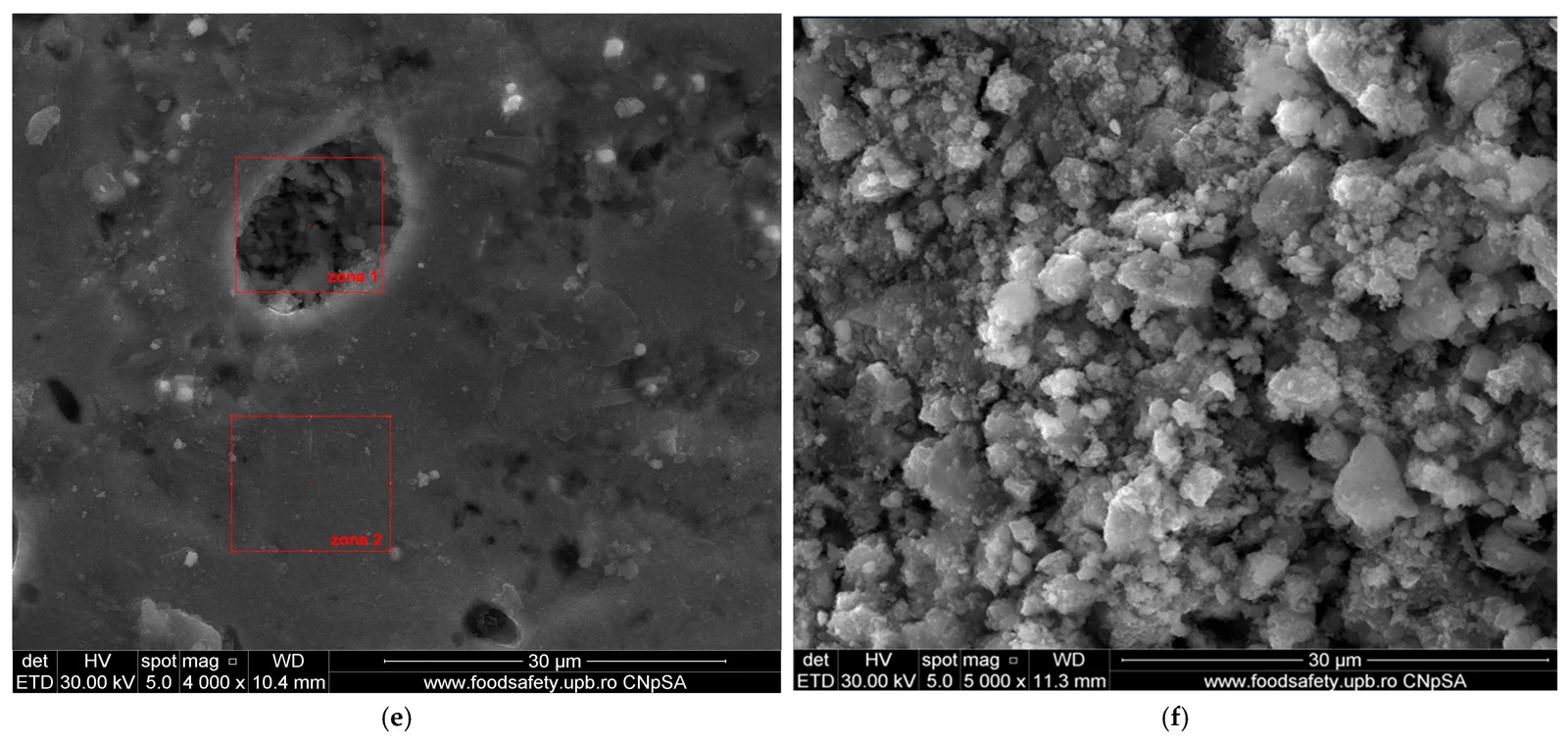
SEM analysis of the inner surface of OLT 37 galvanized pipes, unused and used. (**a**) SEM of unused OLT 37.1 pipe, magnification 200 times, (**b**) SEM of used OLT 37.1 pipe magnification 400 times, (**c**) SEM of unused OLT 37.1 pipe, magnification 2000 times. (**d**) SEM of used OLT 37.1 pipe, magnification 2000 times, (**e**) SEM of unused OLT 37.1 pipe, magnification 4000 times, (**f**) SEM of used OLT 37.1 pipe, magnification 5000 times.

**Figure 12:**
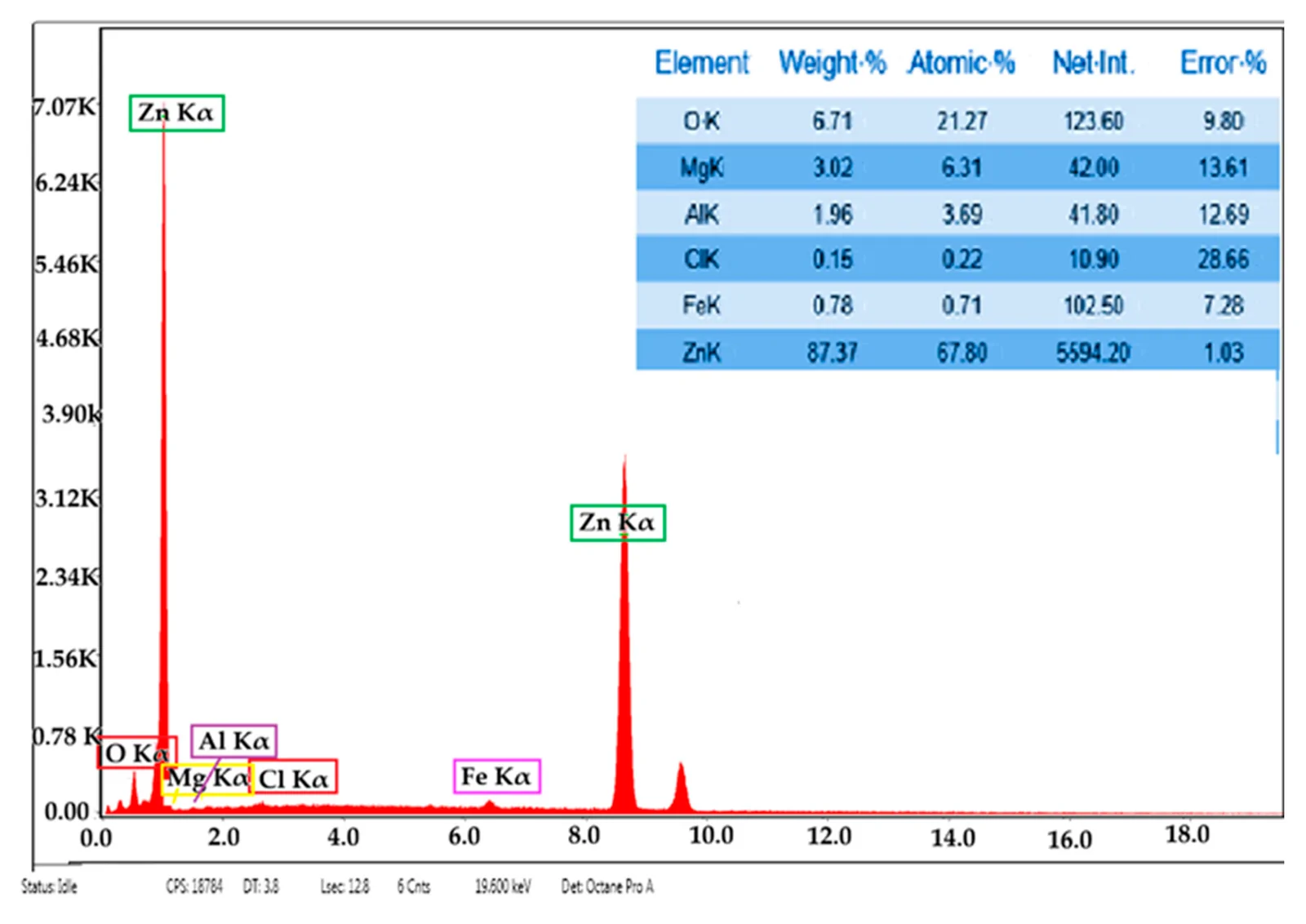
EDX analysis—unused galvanized pipe for Zone 1, corroded area.

The galvanized steel pipes, OLT 37.1. used in transport infrastructure, showed changes in the wall thickness from 3.5 mm initially to 3.2 in areas where zinc is not evident, to 4 mm in areas with deposits in the pipe used for 23 years. The changes that occurred were highlighted by SEM and EDX spectra. From the analysis of the SEM images of the interior of the unused OLT 37 1 pipe, see Figure 11a,c,e, it can be seen that the surface is uniform, with low porosity, with small cavitation zones, which are determined by the non-uniform distribution of the zinc layer on the steel surface. The SEM analysis carried out highlighted the existence of two distinct zones: the presumed cavitation zone corroded in the presence of air, called Zone 1, and the flat, uncorroded zone, called Zone 2, in Figure 11e. Zone 1 is the cavitation zone and extends over an average diameter of 25 µm. From the EDX analysis of Zone 1 (see Figure 12), it was observed that it contains elements such as metallic Zn in a mass concentration of over 87%, but also other elements such as O in a proportion of 6.7%, Mg 3.2%, Al 1.96%, Cl 0.15%, and Fe in a proportion of 0.78%. The superficial zinc layer is predominant but only partially covers the surface and can be in the form of metallic Zn, zinc oxide, or zinc hydroxide. The iron ions present superficially in the cavity can be oxidized to Fe_2_O_3_. Zinc ions in the presence of oxygen from the air form an electrochemical cell in which oxygen is reduced to O^2-^ and zinc is oxidized to form Zn^2+.^ Mg and Al ions can be present as impurities in OLT 37 on which zinc was deposited [35]. These ions can be present in the form of magnesium and aluminum oxides, as identified by Zhang B. et al. [36]. The oxidation process is slow, with the formation of ZnO and, in the presence of atmospheric water, Zn(OH)_2_, which represents a passivating protective layer. The protective layer is stable in the pH range 6.5–12 [37].

**Figure 13:**
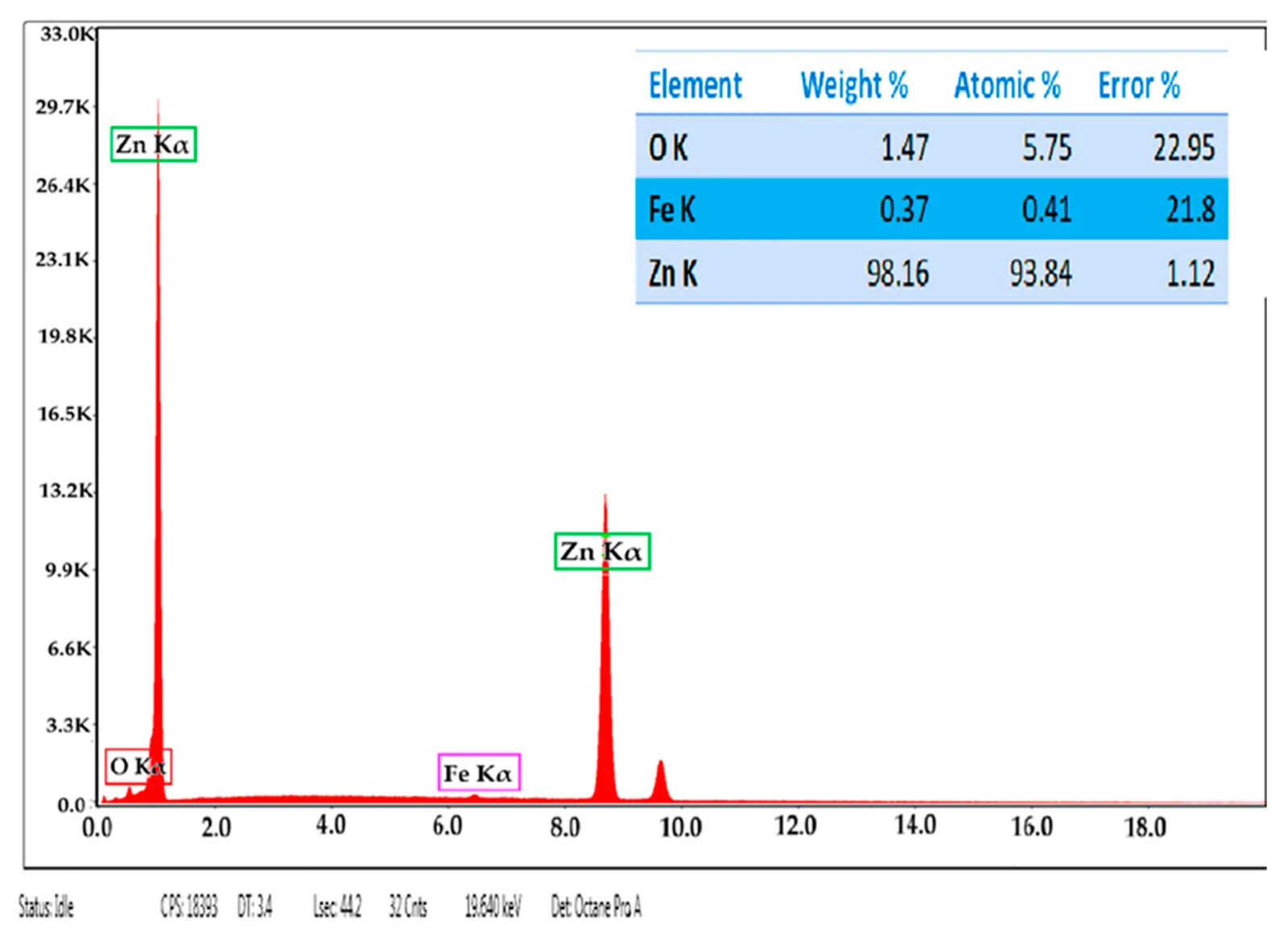
EDX analysis—unused OLT 37.1. pipe for Zone 2, uncorroded area.

From the EDX analysis of the surface of Zone 2, the flat, uniform area see Figure 13, it can be seen that the elements present are metallic Zn in a proportion of over 98%, iron in very low concentrations, which can also be present as oxides, and oxygen being present in concentrations of 1.47%. For almost 12 oxygen atoms, there is 1 iron atom. The superficial zinc layer only partially covers the surface and can be in the form of metallic zinc, zinc oxide, zinc hydroxide, or a passivated layer. In the areas partially covered with zinc, the iron ions are superficial and can be found in the form of metallic iron or in the oxidized form of Fe_2_O_3_ [38].

From the SEM analysis for the galvanized OLT 35 pipe used for a period of 23 years, an inhomogeneous, rough structure with nano- and micro-cavities can be observed at magnifications of 400, 2000, and 5000 times (see Figure 11b,d,f). These cavities can be determined either by the corrosion of the zinc layer, or by deposits of oxides or other salts on the metal surface, or by a combination of both [35].

**Figure 14:**
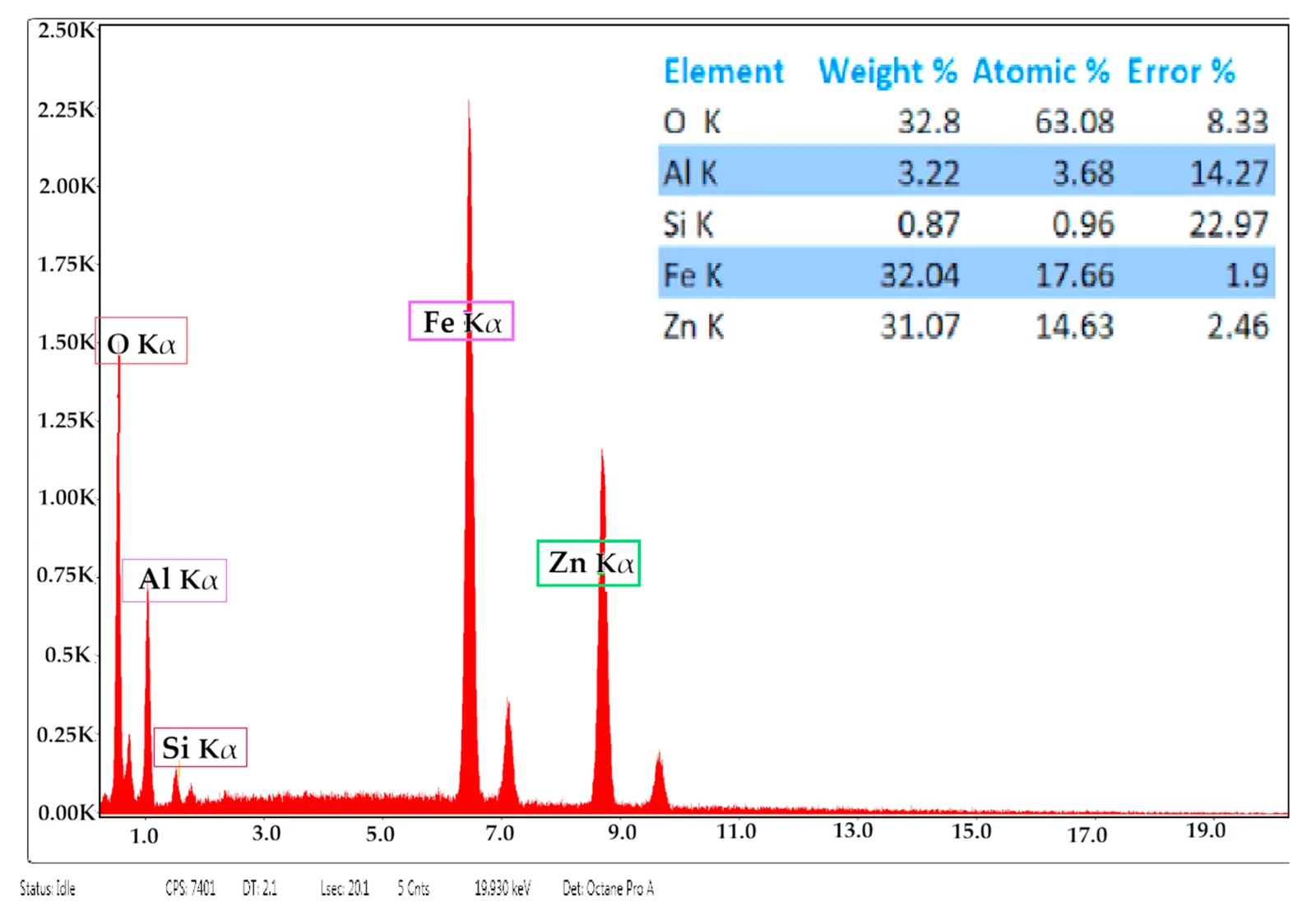
EDX spectrum of the inner surface of the OLT 37.1 pipe used for 23 years.

EDX analysis for these used pipes is presented in Figure 14. It can be seen that the zinc fraction on the surface decreased to 31.07%, and the iron fraction increased to 32.04%. On the internal surface, Al appeared at concentrations of 3.22% and Si at 0.87%, and oxygen increased significantly to 32.8%. Thus, the surface corroded, and the zinc was removed; the iron from the lower layer came to the surface. Zinc can be in the form of metallic zinc, zinc oxide, or zinc hydroxide. A large part of the zinc compounds has been exfoliated and mechanically entrained by the water flow [39]. Silicon, aluminum, calcium, and magnesium from the water have been deposited in these irregularities in the form of oxides or carbonates on the steel surface, as shown in Figure 11f, where microcrystals appear. These can be formed due to different electrochemical potentials that appear at the interface of the pipes between their components: Zn, Fe, Mn, Al, Si, Ca, Mg, H+, during the period of stagnation of the water, etc. [40].

The main reactions that take place on the pipe surface can be:

In aerobic conditions

Anode (pipe)

Fe(s) → Fe^2+^ + 2e^−^

Zn(s) → Zn^2+^ + 2e^−^ (4)

Cathode (water):

O_2_ + 2H_2_O + 4e^−^ → 4OH^−^

In anaerobic conditions:

Anode (pipe)

Fe(s) → Fe^2+^ + 2e^−^

Zn(s) → Zn^2+^ + 2e^−^

Cathode (water):

2H_2_O+2e^−^ → H_2_+2OH^−^

As a result of this reaction, aerobic/anaerobic conditions result in deposits on the pipe of Fe(OH)_2,_ FeOOH, Fe_3_O_4_, ZnS, Zn(OH)_2_, ZnO, FeS, CaCO_3_, SiO_2_, nH2O, Al(OH)_3_, and Mg(OH)_2_, MgCO_3_, CaSiO_3_ [32,33,41].

These deposits protect the pipe from corrosion but reduce the useful diameter of the pipe.

The drinking water transport network has a short section of galvanized pipe located on Luceafarului Street, P3, a pipe with a length of over 400 m and over 19 years old. The comparative results of water characteristic parameters at the station outlet and at the sampling point for the period September-November 2023-2025 can be identified in Table 5. Parameters that undergo changes at the sampling point compared to the station outlet are pH, turbidity, conductivity, free residual chlorine, oxidizability, COD, nitrates, aluminum, and iron. Parameters that do not undergo changes or undergo minor changes are: ammonium, nitrites, chlorides, hardness, sodium, sulfates, and dissolved sulfides. The slight decrease in pH can be attributed, in the first stage, to zinc, which plays the role of anode, the first to dissolve, forming oxides and hydroxides and releasing protons. It is important to know that after the dissolution of zinc, the iron surface is exposed, which, in turn, plays the role of anode and dissolves, forming oxides and releasing protons. In the dissolution of zinc and iron, a special contribution is made by dissolved oxygen, alkalinity, and water hardness.

It is observed that the pH of the water is in the weakly basic range, the hardness is high, tending to ten, optimal conditions for the dissolution of zinc and iron and the deposition of oxides, carbonates and hydroxides of aluminum, silicon, calcium and magnesium, with a protective role on the pipes [42]. This behavior is supported by the fact that the pH of the water at the street consumer decreases, the conductivity increases due to dissolved substances, the hardness decreases slightly due to the deposits of calcium and magnesium ions, aluminum and silicon; the concentration of zinc and iron ions increases due to dissolution in the pipes. The decrease in the residual chlorine concentration and the increase in oxidizability and COD are explicable by the oxidation of organic and inorganic compounds present in the water and are closely related to the increase in turbidity [43]. The analysis of the zinc ions in the street consumer proved that it is not present in the water at concentrations higher than when leaving the station, which is below the limit of quantification (LOQ). Therefore, these pipes older than 23 years no longer release zinc, as seen from SEM, because it is no longer present in the superficial layer; the protective layer has already been solubilized, or other salts have been deposited on them that have passivated the pipe. forming layers of oxides, hydroxides, and carbonates of calcium, magnesium, aluminum, and silicon that protect the pipe and from which particles can be detached, and salts can be dissolved depending on the temperature, pH, pressure, and water flow rate [44].

### 3.4. High-Density Polyethylene, HDPE, Pipes

In Calarasi city, the water and sewer operator that manages the production and distribution of drinking water had as its objective, starting in 2007, the replacement of old HDPE, asbestos cement, OLT 37, and OLT 37.1 pipes with new high-density polyethylene, HDPE pipes. Thus, over 75% of the total drinking water transport and distribution network is made up of polyethylene pipes. In the study conducted, we analyzed SEM and EDX for unused HDPE pipes and those used for over 20 years.

Based on the measurements, it was observed that the HDPE pipes initially had a wall thickness of 5 mm. After 20 years of use, areas were identified where the thickness varied from 4.9 mm in corrosion areas to areas with deposits where the diameter reached 5.2 mm. This is a fortunate case, determined by a not-too-high hardness. There are cases where deposits on HDPE can reach a size on the order of tens of mm. The changes that occurred were highlighted by SEM and EDX spectra.

**Figure 15:**
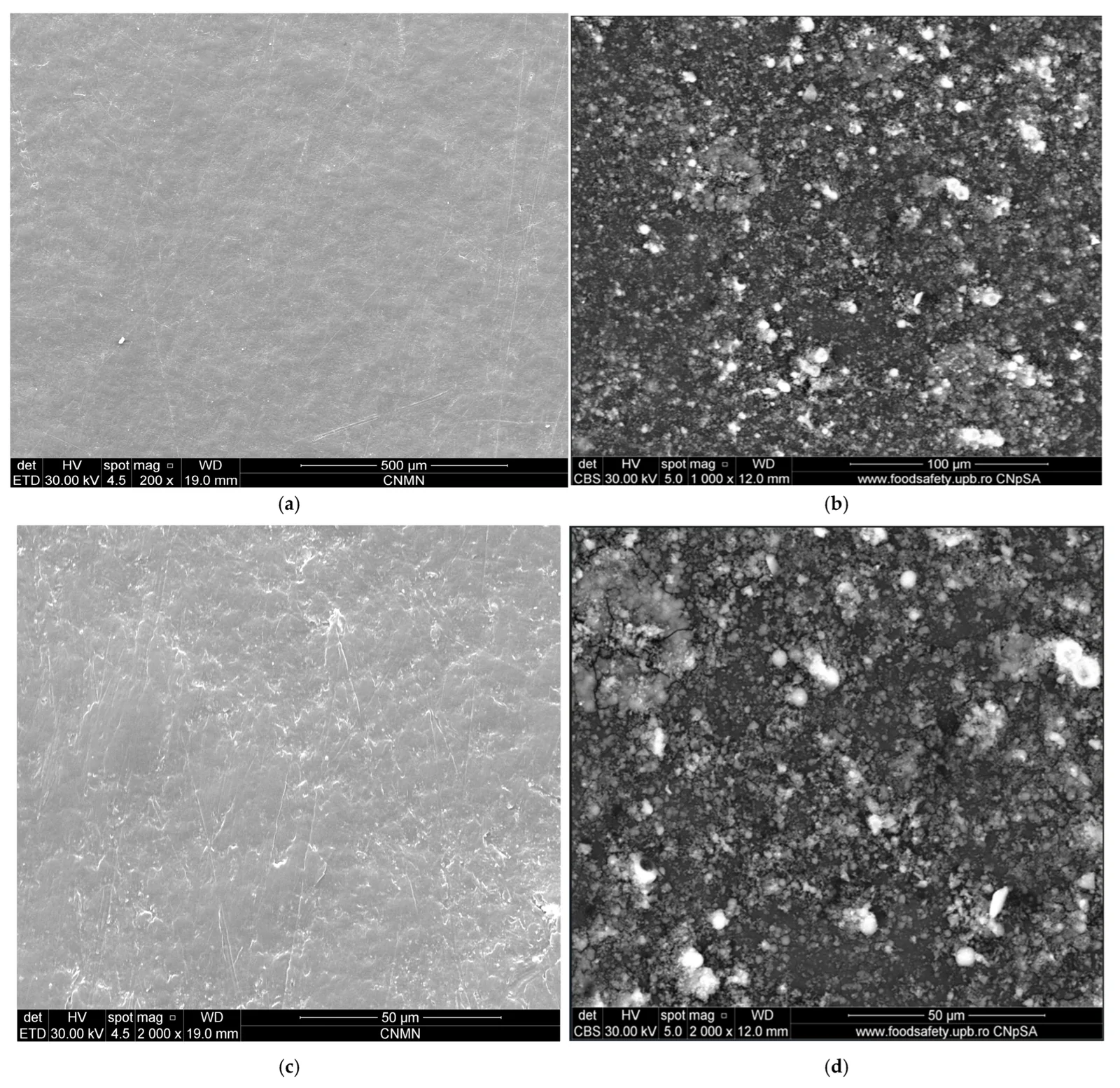

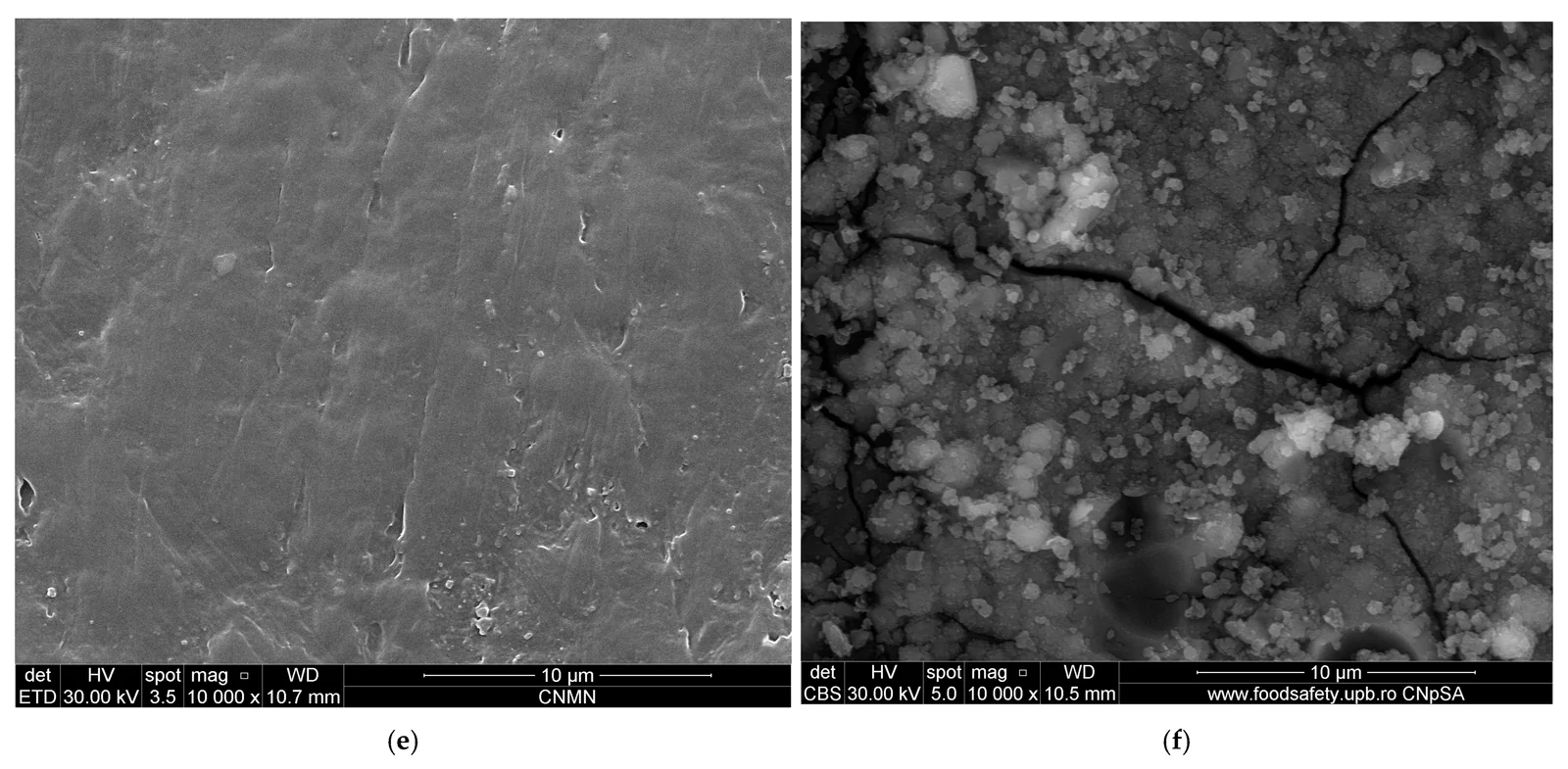
SEM analyses of the inner surface of HDPE pipes, unused and used. (**a**) SEM of the unused HDPE pipes, magnification 200 times, (**b**) SEM of the used HDPE pipes, magnification 200 times, (**c**) SEM of the unused HDPE pipes, magnification 2000 times, (**d**) SEM of the used HDPE pipes, magnification 2000 times, (**e**) SEM of the unused HDPE pipe, magnification 10,000 times, (**f**) SEM of the used HDPE pipe, magnification 10,000 times.

From the SEM analysis of unused HDPE pipes (see Figure 15a,c,e), it can be seen that the internal surface of the pipe is relatively uniform, without major unevenness or deposits, and without fractures or cracks. The SEM image was obtained after depositing a thin layer of gold on the surface.

After long-term use, the appearance of the pipe has changed radically, as shown in Figure 15b,d,f. The surface is non-homogeneous with microcrystalline and amorphous deposits of different shapes, sizes, and shades. The dimensions of the deposits are in the micrometer range.

**Figure 16:**
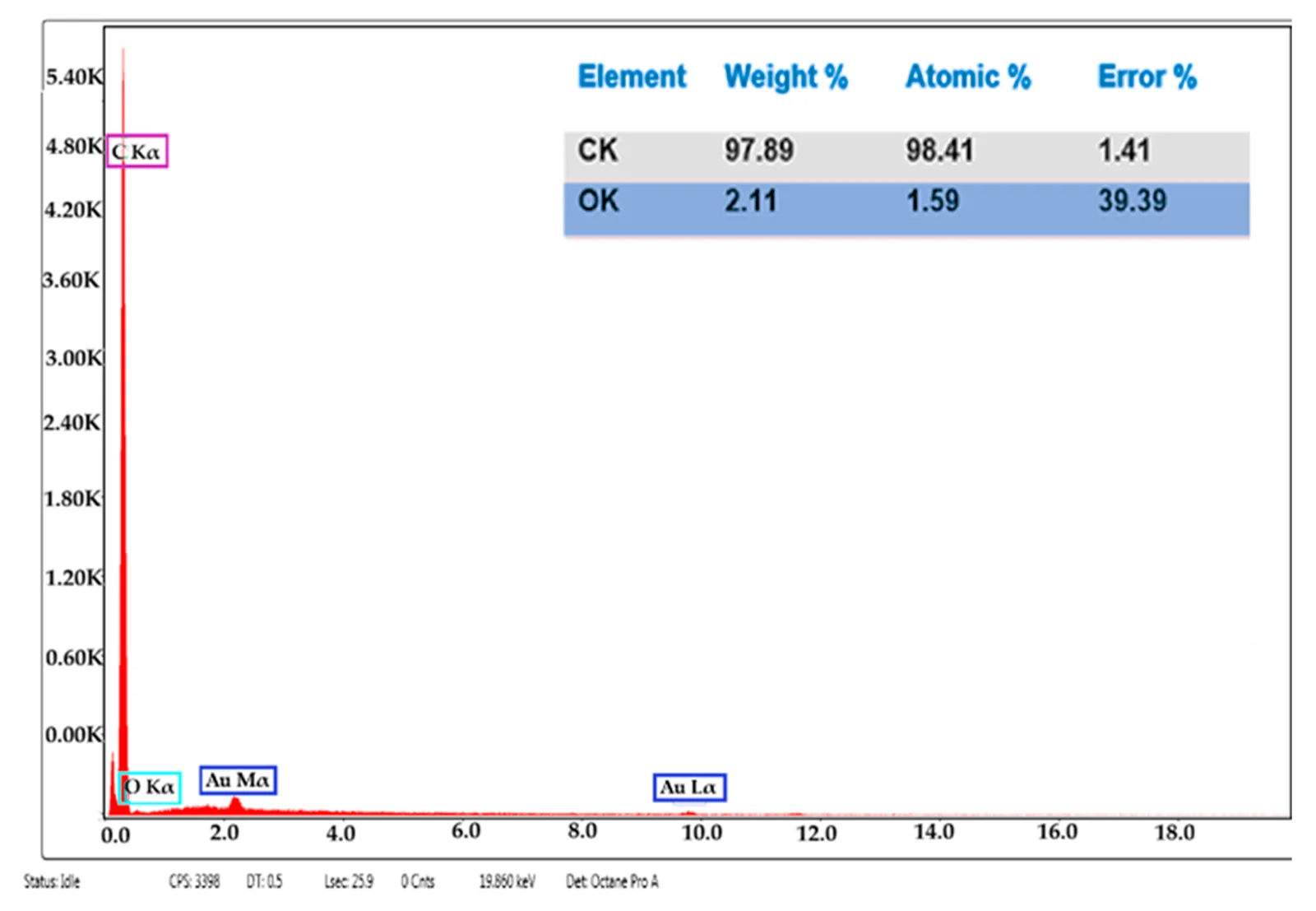
EDX spectrum of the inner surface of the unused HDPE pipe and the percentage of the main elements identified.

From the analysis of the EDX spectrum presented in Figure 16, it can be seen that the internal surface of the unused HDPE pipe is made up of two carbon elements in a proportion of up to 98% and oxygen in a concentration of around 2%. The presence of oxygen suggests some hydration of the superficial layer in the presence of water. In HDPE, in the presence of O_2_ and ClO_2_ from water, hydration of the polymer takes place, followed by its oxidation. Free chlorine, chloramines, and chlorine dioxide can initiate oxidative reactions that progressively consume antioxidant stabilizers and promote chain scission, leading to changes in mechanical, thermal, and structural properties of HDPE [45,46]. The presence of free radicals, P•, used to initiate the polymerization process and unsaturation in the polymer composition can favor this process. They can form hydroperoxyl, POO•, which can further decompose into oxyl and hydroxyl radical groups, HO•, which favor the fragmentation of the polymer and the possible formation of microplastics. Also, the mechanical stress generated by the water pressure in the flow process favors the degradation process by friction [47]. The processes that can take place are presented below [48].

P• + O_2_→POO• (5)

POO• PH → POOH + P•(6)

POOH → PO• + HO•(7)

The degradation process of HDPE pipes is a very slow process that begins upon exposure to sunlight (visible, ultraviolet, and infrared light), before integration into the water supply system. It is recommended to store them in a place with as little time as possible in strong light and with as small day-night and winter-summer temperature variations as possible.

The degradation process of HDPE pipes intensifies following their introduction into the water transport and supply system, in the presence of pressure shocks, the chemical composition of the water, the presence of dissolved oxygen, and dissolved salts. The phenomenon is called slow crack growth [49].

**Figure 17:**
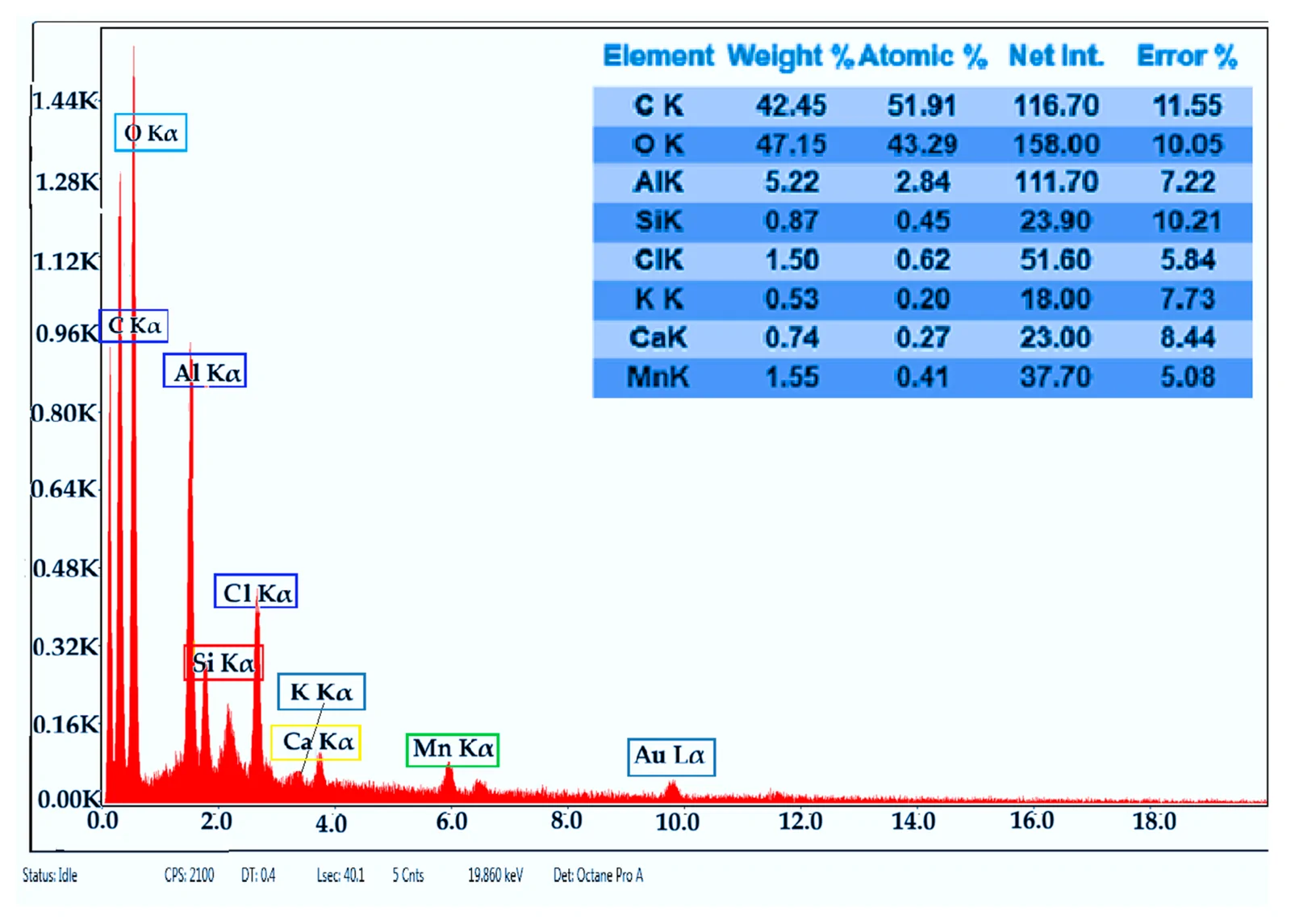
EDX spectrum of the inner surface of the used HDPE pipe for 20 years and the percentage of the main elements identified.

**Figure 18:**
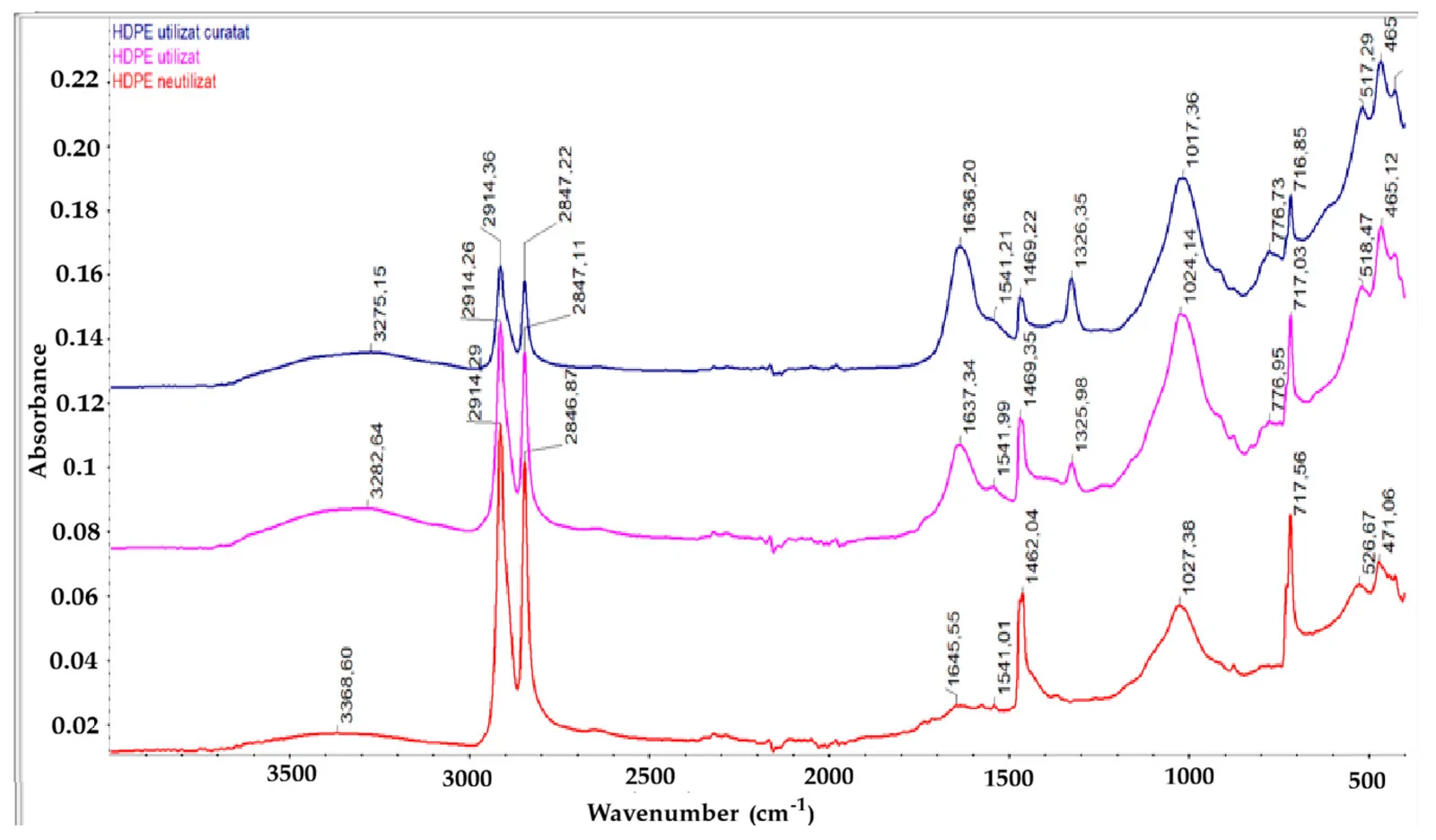
Comparation between FTIR Spectrum of the unused and used cleaning of deposits HDPE pipes.

After prolonged contact with water and mechanical stress, HDPE pipes show crystalline and amorphous deposits but also microcracks, as expected, as can be seen in Figure 15b,d,f. From the EDX spectrum analysis in Figure 17 for the used HDPE pipe, it can be seen that the deposits consist mainly of aluminum oxides but also of silicon and manganese, calcium carbonates, and chlorides. Of course, on the surface of the HDPE pipe, carbon is found in concentrations of 42%. A result that highlights corrosion is the fact that oxygen reaches a percentage of 47%, much too high to be only oxides and hydroxides. The increase in oxygen concentration may be due to the deposition on the surface of the HDPE pipe of oxides or carbonates such as CaCO_3_, Mg(OH)_2_, MnO_2_, Al(OH)_3_, SiO_2_, nH_2_O, etc., but also to processes that target HDPE such as hydration and oxidation, as can be seen in Figure 18. An important role in this process is also played by free chlorine, especially in the form of Cl_2_O [50,51]. Since the pipe was replaced after 20 years of use, we can understand that the deterioration process of the HDPE polymer began before installation in the supply system and continued, little by little. After 20 years of use, deep microcracks appeared, leading to the gradual grinding of the pipe but also to deposits on the surface that influence the turbidity and conductivity of the water through solubilization and precipitation, depending on the operating conditions. The water analysis was carried out on Sulfinei Street, point P4 (see Figure 5). The transport infrastructure is made of HDPE only; the total section is approximately 800 m from the WTS. Sampling point P4 is located approximately 650 m from the station departure.

The results of the water analysis are presented in Table 6. A slight increase in the pH of the water from the station outlet to the street consumer can be observed, with values between 7 and 7.5, because chlorine gas is consumed, which forms hypochlorite, causing an increase in pH. Hypochlorite is consumed by the oxidation of organic compounds, HDPE pipes, and microorganisms [52,53,54]. Residual chlorine decreases due to oxidation processes that take place on the chlorine-containing pipe.

The turbidity of the water at the consumer is higher than at the station outlet in all sampling periods. These additional particles may be particles detached from the pipes and/or be trivalent iron hydroxide or aluminum oxides or dead microorganisms. The conductivity of the water increases relatively little due to the solubilization of some salts from deposits due to day-night thermal variations. Ammonium and nitrite are below the limit of quantification; nitrates decrease very little in the pipeline, possibly due to their consumption in the pipeline by possible microorganisms. Oxidizability variables from one year to another; it has decreased over time. At the same time, the same trend of decreasing manganese and iron concentrations can be observed. This suggests that oxidizability is related to the concentration of these ions, which can be found in the reduced state of manganese and bivalent iron and can be oxidized to trivalent iron and tetravalent manganese at the same time, causing an increase in turbidity and deposits on the pipeline, an idea also supported by Kohl and Medlar [55]. The aluminum ion decreases as a concentration in the water due to its deposition on the pipeline in the form of aluminum oxide. Hardness also decreases slightly due to calcium deposits in the form of carbonates, hydroxides, or sulfates. The concentration of chloride ions increases due to the transformation of hypochlorite into chlorides. The concentrations of sodium and sulfate ions do not change in water.

From the analysis of the FTIR spectra for unused and used cleaning of deposits HDPE pipes, shown in Figure 18, the common band wavenumbers [56,57] can be seen. The wavenumbers at 2914 cm^−1^ and 2847 cm^−1^ are attributed to the (CH_2_) group asymmetric and symmetric stretching; at 1462–1469 cm^−1^ attributed to δ(CH_2_) bending (deformation); at 1024–1027 cm^−1^ attributed to C- O type vibrations and oxidation products; and 717 cm^−1^ attributed to CH_2_ rocking, characteristic of the semi-crystalline structure of long polyethylene chains. These bands are common to the IR spectra of HDPE and confirm the maintenance of the basic structure of the polymer in all the analyzed samples. A decrease in the height of all characteristic HDPE peaks is observed from the unused material to the used one and the used one with cleaning of deposits, which may indicate a structural modification of the polymer chains due to aging and oxidation [56,58].

Also, bands can be observed that show differences between the unused, used and used cleaning of deposit HDPE pipes, namely in the wavenumber range of: 3400–3300 cm^−1^ broad band centered around the values 3386–3275 cm^−1^ which is attributed to the adsorbed water molecule, hydroxyl groups (-OH), and polyethylene oxidation products (alcohols, hydroperoxides); this band is much increased in the case of the used and used cleaning of deposit HDPE pipes. The intensification of this band in the used and used-cleaned samples suggests an increase in the polarity of the material surface as a result of oxidative aging [56,57,58].

The wavenumber range between 1600 and 1800 cm^−1^, in our case between 1645 and 1634 cm^−1^, is attributed to the characteristic vibrations of carbonyl groups, C=O and unsaturation, C=C bonds. The absence of a pronounced carbonyl band in the 1710–1740 cm^−1^ region does not allow the assertion of the existence of advanced oxidative degradation; carbonyl groups represent the main FTIR marker of polyethylene oxidation [56,57,58]. The peak at 1645 cm^−1^, characteristic of the unused pipe, is almost insignificant in the unused pipe and increases in height and width from the used pipe to the used pipe cleaned of deposits. This proves that there are changes in the structure of older pipes. This behavior of the pipes is also confirmed by the SEM image of the used pipe (see Figure 15).

## 4. Conclusions

The materials of the pipes used for the distribution of drinking water in the city of Calarasi are asbestos cement (very few), OLT 37 (over 22% of the total network), and OLT 37.1. (very few), with HDPE over 76%. The drinking water transport infrastructure that was outdated, with a lifespan of 20 to 46 years, was partially replaced with high-density polyethylene pipes. It was found that the asbestos cement pipes were corroded by partial grinding of the cement and the exposure of fibrous asbestos with a high potential for fragmentation into fine particles, aluminosilicate microfibers, which are toxic. The lifespan of the asbestos cement pipes exceeded 46 years. It would be interesting to study how long it takes for asbestos fibers to appear. In the water at the consumer end, an increase in pH, turbidity, conductivity, and oxidability is observed due to the detachment of particles and the dissolution of salts from the cement. These pipelines are included in the Very High/Critical risk category [59,60].

From the analysis of the OLT 37 pipes, it was found that they corrode and grind; iron ions and other steel components are released from the pipes, and other compounds are deposited on the pipes, forming irregular deposits of oxides, carbonates, sulfates, hydroxides of calcium, iron, manganese, silicon and aluminum. The surface of the deposits is covered in uniform, acicular microcrystals ranging in size from micrometers to hundreds of nanometers that protect the surface. The development of these structures occurs in a pipe used for over 42 years.

In the water transported on the OLT 37 pipeline, a sharp increase in oxidability, conductivity, and turbidity is observed due to the oxidation of iron and manganese ions, their solubilization in the first stage and then advanced oxidation to trivalent iron and tetravalent manganese, present either in a dissolved state or as particles that give toxicity to the water. These pipelines from OLT 37 are classified as medium risk [32,61].

In the OLT 37.1 pipelines, a uniform layer of zinc hydroxide is formed in the first stage, protecting the steel layer. Zinc corrodes in the presence of oxygen from the air but also oxygen from the water. Since an electrochemical cell is formed in which oxygen is reduced and zinc is oxidized, zinc oxides are formed and then zinc hydroxide, a passivating protective layer. Pipes fabricated from OLT 37.1 exhibit slightly improved mechanical performance and durability than OLT 37. The risk category for such pipes may therefore be assigned to a low-to-moderate [32,61].

The main degradation mechanism for both steel materials remains electrochemical corrosion, particularly under aggressive environmental conditions. Problems arise in such pipelines when the zinc layer is not uniform, and areas appear where it is missing. This is how the specific corrosion of OLT 37 occurs. On the galvanized pipe used for over 23 years, the disappearance of the zinc layer can be observed due to the different electrochemical potentials of the pipe components and the specific corrosion of OLT 37. The water quality is influenced by these processes, and changes in pH, turbidity, conductivity, oxidizability, aluminum, and iron occur as in the case of OLT 37. After the disappearance of zinc, the behavior of the pipe is similar to that of OLT 37.

The high-density polyethylene (HDPE) pipes were replaced after 20 years of use. Problems related to the gradual embrittlement and aging of the material in the presence of oxygen and chlorine in the water can be observed. Also, deposits of microcrystals and amorphous substances of oxides, hydroxides, and carbonates are formed on the surface of the pipe. Water quality is modified compared to the exit from the station by the increase in turbidity determined by the microplastics that are released by the fractionation of the iron and manganese oxide particles in the pipe; of conductivity, oxidizability, and iron, manganese, and aluminum concentrations. Although water quality at the monitored street points falls within the maximum permissible limits (MPL), changes in its quality can be observed, but within the limits imposed by law. At this moment, HDPE pipes are classified as low risk [61,62].

As a recommendation regarding the use of HDPE pipes in water distribution systems intended for human consumption, the concentration of chlorine compounds used in disinfection processes should be reduced; and polyethylene material to contain a sufficient type and number of antioxidants to protect the polymer from oxidative degradation during manufacturing, storage, and long-term service.

The quality of water changes depending on the material of which the transport pipe is made, and the long-term use of water with slightly modified characteristics but with toxicity can be determined by asbestos microfibers, iron and manganese oxides, zinc, and fine particles (microplastics) of HDPE, which can influence the health of the population. Changes in pipes occur after a shorter or longer period of use; it is noteworthy that the asbestos cement AC and OLT 37 pipes last over 42 years, while the HDPE pipes fail quite quickly due to cracks and fragmentation after only 20 years of use.

A ranking according to the risk category for the materials from which the analyzed pipes are made can be expressed as follows:

AC > OLT 37 > OLT 37.1 > HDPE

HDPE pipes currently have the highest level of operational safety.

## Figures and Tables

**Table 1:** Water sampling points from the drinking water supply network, street consumers located on public property.

No. crt.	Sampling Point	Pipeline Characteristics	Street Length, m/Distance Traveled by Water, m
WTS	Exit water treatment plant, WTS, Eroilor Street	Point zero, witness	0
P1	Nufarului Street	Pipe with a diameter of 200 mm, asbestos cement, 44 years old	631/453: the last 55 m is asbestos cement
P2	Belsugului Street	Pipe size 200 mm, steel OL37, 42 years old	1237/720
P3	Luceafarului Street	Galvanized steel, with diameters of 50 and 90 mm, 23 years old.	2475/2150: the last 410 m is galvanized steel pipe
P4	Sulfinei Street	Variable-size pipe, high-density polyethylene with a diameter of 160 mm, 20 years old	1385/1156

**Table 2:** Analyzed parameters, measurement uncertainties, and standard methods used.

No. Crt.	Parameter	Unit Measurement	Measurement Uncertainty	Analysis Method
1	pH (25 °C)	unit	±0.1312 unit. pH	SR EN ISO 10523:2012
2	Turbidity	NTU	±0.060 NTU	SR EN ISO 7027/2001
3	Conductivity (25 °C)	µS/cm	±13.3 µS/cm	SR EN 27888/1997
4	Free residual chlorine	mg/L	±0.02 mgCl_2_/L	SR EN ISO 7393-2/2018
5	Ammonium	mg/L	±0.001 mg NH_4_/L	SR ISO 7150-1/2001
6	Nitrites	mg/L	±0.001 mg NO_2_/L	SR ISO 7890-3:2000
7	Nitrates	mg/L	±0.10 mgNO_3_/L	SR EN ISO 26777:2002
8	COD (oxidizability)	mg O_2_/L	±0.07 mgO_2_/L	SR EN ISO 8467/2001
9	Chlorine	mg/L	±1.08 mg/L	SR ISO 9297/2001
10	Total hardness	°G	±0.20 °G	SR ISO 6059/2008
11	Iron	μg/L	±2.529 µg Fe/L	SR13315:2008; SRENISO15586:2008
12	Manganese	μg/L	±2.521 µg Mn/L	SR 8662-2/1997
13	Aluminum	µg/L	±2.520 µg Al/L	SRENISO12020:2008
14	Sodium	mg/L	±2.521 mgNa/L	STAS3223/2/1980
15	Sulphats	mg/L	±1.46 mg SO_4_/L	STAS 3069/87
16	Solved Sulphurs	μg/L	±1.30 µg 1/L	SR ISO10530/1997

**Table 3:** Characteristics of drinking water at the street consumer, P1, Street Nufarului, compared to the control, Water Treatment Station, WTS.

No. Crt.	Parameter	Unit Measurement	WTS, 21 September 2023	P1, 21 September 2023	WTS, 22 August 2024	P1, 22 August 2024	WTS, 22 September 2025	P1, 22 September 2025	LV ^1^
1	pH (25 °C)	unit	7.515	7.864	7.051	7.560	7.450	7.952	6.5–9.5
2	Turbidity	NTU	0.13	0.293	0.280	0.210	0.210	0.360	
3	Conductivity (25 °C)	µS/cm	440	587	347	463	320	460	2500 at 20 °C
4	Free residual chlorine	mg/L	0.48	0.36	0.30	0.22	0.51	0.43	0.1–0.5
5	Ammonium	mg/L	<LOQ	<LOQ	<LOQ	<LOQ	<LOQ	<LOQ	0.5
6	Nitrites	mg/L	<LOQ	<LOQ	<LOQ	<LOQ	<LOQ	<LOQ	50
7	Nitrates	mg/L	5.3	5.28	4.900	5.000	4.700	4.780	0.1
8	COD	mg O_2_/L	0.861	1.263	0.344	0.875	0.174	0.521	5
9	Chlorine	mg/L	23.075	23.07	27.974	27.980	25.989	26.99	250
10	Total hardness	°G	9.159	9.157	8.791	8.790	8.911	8.910	≥5
11	Iron	μg/L	21.17	22.32	13.900	14.840	16.180	16.980	200
12	Manganese	μg/L	0.913	0.934	0.249	0.258	2.071	2.320	50
13	Aluminum	µg/L	186.8	182.21	191.50	188.30	188.50	172 8	200
14	Sodium	mg/L	9.66	9.62	14.32	14.24	13.08	13.13	200
15	Sulphats	mg/L	24	22.7	25.0	24.5	24.0	23.9	250
16	Solved Sulphurs	μg/L	<LOQ	<LOQ	<LOQ	<LOQ	<LOQ	<LOQ	100

^1^ Limit values according to European Directive 202/2184 for water intended for human consumption.

**Table 4:** Characteristics of drinking water at street consumer P2, Strada Belsugului, compared to the control point, Water Treatment Station (WTS).

No. Crt.	Parameter	Unit Measurement	WTS, 21 September 2023	P1, 21 September 2023	WTS 22 August 2024	P1, 22 August 2024	WTS 22 September 2025	P1, 22 September 2025	LV ^1^
1	pH (25 °C)	unit	7.515	7.56	7.051	7.060	7.450	7.500	6.5–9.5
2	Turbidity	NTU	0.13	0.21	0.280	0.291	0.210	0.207	0.5
3	Conductivity (25 °C)	µS/cm	440	454	347	393	320	355	2500 la 20 °C
4	Free residual chlorine	mg/L	0.48	0.12	0.30	0.12	0.51	0.44	0.1–0.5
5	Ammonium	mg/L	<LOQ	<LOQ	<LOQ	<LOQ	<LOQ	<LOQ	0.5
6	Nitrites	mg/L	<LOQ	<LOQ	<LOQ	<LOQ	<LOQ	<LOQ	0.1
7	Nitrates	mg/L	5.3	5.4	4.900	5.000	4.700	4.800	50
8	COD	mg O_2_/L	0.861	1.021	0.344	1.071	0.174	0.348	5
9	Chlorine	mg/L	23.075	23.085	27.974	27.934	26.02	25. 99	250
10	Total hardness	°G	9.159	9.147	8.791	8.62	8.911	8.78	≥5
11	Iron	μg/L	21.17	23.16	13.900	14.25	16.180	16.431	200
12	Manganese	μg/L	0.913	1.85	0.249	0.476	2.071	2.453	50
13	Aluminum	µg/L	186.8	167	191.50	167.00	188.50	178.10	200
14	Sodium	mg/L	9.66	9.58	14.32	14.23	13.08	13.18	200
15	Sulphats	mg/L	24	23.88	25.0	24.97	24.0	24.9	250
16	Solved Sulphurs	μg/L	<LOQ	<LOQ	<LOQ	<LOQ	<LOQ	<LOQ	100

^1^ Limit values according to European Directive 202/2184 for water intended for human consumption.

**Table 5:** Characteristics of drinking water at the street consumer point P3, Luceafarului Street, compared to those at the control point exiting the station, WTS.

No. Crt.	Parameter	Unit Measurement	WTS, 21 September 2023	P1, 21 September 2023	WTS 22 August 2024	P1, 22 August 2024	WTS 22 September 2025	P1, 22 September 2025	LV ^1^
1	pH (25 °C)	unit	7.515	7.426	7.051	7.032	7.450	7.610	6.5–9.5
2	Turbidity	NTU	0.13	0.35	0.280	0.330	0.210	0.320	
3	Conductivity (25 °C)	µS/cm	440	469	347	385	320	329	2500 la 20 °C
4	Free residual chlorine	mg/L	0.48	0.2	0.30	0.16	0.51	0.24	0.1–0.5
5	Ammonium	mg/L	<LOQ	<LOQ	<LOQ	<LOQ	<LOQ	<LOQ	0.5
6	Nitrites	mg/L	<LOQ	<LOQ	<LOQ	<LOQ	<LOQ	<LOQ	0.1
7	Nitrates	mg/L	5.3	5.1	4.900	4.7	4.700	4.54	50
8	COD	mg O_2_/L	0.861	0.851	0.344	1.362	0.174	0.356	5
9	Chlorine	mg/L	23.075	23.072	27.974	27.980	25.989	25.989	250
10	Total hardness	°G	9.159	9.14	8.791	8.782	8.911	8.903	≥5
11	Iron	μg/L	21.17	23.32	13.900	15.490	16.180	18.060	200
12	Manganese	μg/L	0.913	0.929	0.249	0.252	2.071	2.153	50
13	Aluminum	µg/L	186.8	156.7	191.50	165.00	188.50	158.90	200
14	Sodium	mg/L	9.66	9.64	14.32	14.40	13.08	13.08	200
15	Sulphats	mg/L	24	24	25.0	25.2	24.0	24.0	250
16	Solved Sulphurs	μg/L	<LOQ	<LOQ	<LOQ	<LOQ	<LOQ	<LOQ	100

^1^ Limit values according to European Directive 202/2184 for water intended for human consumption.

**Table 6:** Characteristics of drinking water at the street consumer point P4, Sulfinei Street, compared to those at the control point exiting the station, WTS.

No. Crt.	Parameter	Unit Measurement	WTS, 21 September 2023	P1, 21 September 2023	WTS 22 August 2024	P1, 22 August 2024	WTS 22 September 2025	P1, 22 September 2025	LV ^1^
1	pH (25 °C)	unit	7.515	7.54	7.051	7.130	7.450	7.570	6.5–9.5
2	Turbidity	NTU	0.13	0.27	0.280	0.328	0.210	0.245	
3	Conductivity (25 °C)	µS/cm	440	441	347	350	320	320	2500 la 20 °C
4	Free residual chlorine	mg/L	0.48	0.18	0.30	0.23	0.51	0.51	0.1–0.5
5	Ammonium	mg/L	<LOQ	<LOQ	<LOQ	<LOQ	<LOQ	<LOQ	0.5
6	Nitrites	mg/L	<LOQ	<LOQ	<LOQ	<LOQ	<LOQ	<LOQ	0.1
7	Nitrates	mg/L	5.3	5.2	4.900	4.600	4.700	4.600	50
8	CCOMn	mg O_2_/L	0.861	0.851	0.34	0.327	0.174	0.154	5
9	Chlorine	mg/L	23.075	23.086	27.974	27.985	25.989	25.997	250
10	Total hardness	°G	9.159	9.146	8.791	8.682	8.911	8.876	≥5
11	Iron	μg/L	21.17	21.13	13.900	13.82	16.180	16.130	200
12	Manganese	μg/L	0.913	0.910	0.249	0.250	2.071	2.073	50
13	Aluminum	µg/L	186.8	160.0	191.50	163.50	188.50	174.20	200
14	Sodium	mg/L	9.66	9.68	14.32	14.31	13.08	13.05	200
15	Sulphats	mg/L	24	24	25.0	25.0	24.0	24.0	250
16	Solved Sulphurs	μg/L	<LOQ	<LOQ	<LOQ	<LOQ	<LOQ	<LOQ	100

^1^ Limit values according to European Directive 202/2184 for water intended for human consumption.

## Data Availability

The original contributions presented in this study are included in the article. Further inquiries can be directed to the corresponding authors.
